# The presence of *Anf/Hesx1* homeobox gene in lampreys suggests that it could play an important role in emergence of telencephalon

**DOI:** 10.1038/srep39849

**Published:** 2016-12-23

**Authors:** Andrey V. Bayramov, Galina V. Ermakova, Fedor M. Eroshkin, Alexandr V. Kucheryavyy, Natalia Y. Martynova, Andrey G. Zaraisky

**Affiliations:** 1 Shemyakin-Ovchinnikov Institute of Bioorganic Chemistry, Russian Academy of Sciences, Moscow, 117997, Russia; 2 Severtsov Institute of Ecology and Evolution, Russian Academy of Sciences, Moscow, 119071, Russia

## Abstract

Accumulated evidence indicates that the core genetic mechanisms regulating early patterning of the brain rudiment in vertebrates are very similar to those operating during development of the anterior region of invertebrate embryos. However, the mechanisms underlying the morphological differences between the elaborate vertebrate brain and its simpler invertebrate counterpart remain poorly understood. Recently, we hypothesized that the emergence of the most anterior unit of the vertebrate brain, the telencephalon, could be related to the appearance in vertebrates’ ancestors of a unique homeobox gene, *Anf/Hesx1*(further *Anf*), which is absent from all invertebrates and regulates the earliest steps of telencephalon development in vertebrates. However, the failure of *Anf* to be detected in one of the most basal extant vertebrate species, the lamprey, seriously compromises this hypothesis. Here, we report the cloning of *Anf* in three lamprey species and demonstrate that this gene is indeed expressed in embryos in the same pattern as in other vertebrates and executes the same functions by inhibiting the expression of the anterior general regulator *Otx2* in favour of the telencephalic regulator *FoxG1*. These results are consistent with the hypothesis that the Anf homeobox gene may have been important in the evolution of the telencephalon.

One of the most important innovations of vertebrates, distinguishing them from other animals, is their complex brain, derived from three main embryonic units: the forebrain, the midbrain and the hindbrain^1^,^2^. It has recently been shown that the core genetic mechanisms regulating the early patterning of the brain rudiment in vertebrates are very similar to those operating during the development of the anterior region of invertebrate embryos^1^,^3^,^4^,^5^,^6^. However, the mechanisms underlying the obvious differences between the vertebrate brain and its invertebrate homologues are still poorly understood.

In 1992, we identified a previously unknown homeobox gene, named *Xanf*, based on its expression in the anterior fold of the neural plate of *Xenopus laevis* embryos, and we then described *Xanf* orthologues in other species of vertebrates, including humans^7^,^8^,^9^,^10^,^11^. Thus, a novel monogenic class of homeoboxes, designated *Anf*, was described^9^. Moreover, an *Anf* orthologue was identified in mice by two other research groups and was designated *Hesx1* or *Rpx*, after its expression in mouse ES cells and in the Rathke pouch^12^,^13^,^14^.

Although *Anf* genes have been discovered in members of most classes of vertebrates, no orthologues of these genes have been found in invertebrates, even in the closest relatives of vertebrates, the invertebrate chordates. Importantly, this distribution correlates with the presence of the telencephalon in vertebrates, which is a unique region of the forebrain that is apparently absent in all other animals and is derived from the anterior neural fold, where *Anf* is expressed. Accordingly, gain- and loss-of-function experiments performed in *Xenopus* and mouse models have confirmed an essential role of *Anf* homeodomain proteins in the development of the telencephalon^10^,^11^,^13^. Thus, in mouse *Anf/Hesx1*^−/−^ mutants telencephalic vesicles and eyes are reduced or absent at early somite stages^15^. Furthermore, using a *Xenopus* model, we have demonstrated that *Anf* acts as a transcriptional repressor, and its main function is “cleaning” the prospective rostral forebrain territory of *Otx2* homeobox expression, which normally regulates the development of more posterior brain regions^11^. As a result, genes responsible for telencephalon development, such as *FoxG1*, can become activated within this territory that has been “cleaned” of Otx2.

On the basis of all of these data, we hypothesized that the appearance of the *Anf* homeobox at the very beginning of vertebrate evolution could be one of the critical events that provided appropriate conditions for the appearance of the telencephalon^11^. However, the failure to detect *Anf* hitherto in the most basal group of extant vertebrates, the cyclostomes, including lampreys and hagfishes, seriously compromised this hypothesis; it began to seem even more questionable after the recent publication of the complete lamprey *Petromyzon marinus* genome, in which no *Anf* orthologues were revealed^16^. Nevertheless, to finally test our hypothesis, we have now made one more attempt to identify this gene in lamprey. As a result, we cloned *Anf* in this animal and demonstrated that this homeobox gene is indeed expressed in the same pattern as in other vertebrates and executes the same functions (by) inhibiting the expression of *Otx2* and promoting the expression of the telencephalic marker gene, *FoxG1*. These results indicate that Anf likely emerged at the beginning of vertebrate evolution and may have been essential for the evolution of the telencephalon.

## Results

### Cloning of *Anf* in lampreys

To clone a possible *Anf* orthologue in the lamprey, we used a previously elaborated procedure for the mechanical enrichment of putative *Anf* transcripts^9^. To this end, we extracted total RNA from the head protrusions of *L. camtschaticum* and *L. fluviatilis*, cut at stage 20–21^17^, and prepared a PCR cDNA library based on this RNA (see Materials and Methods for details). As *Anf* is only expressed in the anterior neural fold in all gnathostomes studied to date, we employed this method because of its enrichment of *Anf* transcripts compared with RNA isolated from whole embryos. This approach also helped us to reduce the concentrations of undesirable transcripts of other homeobox genes, which are not expressed within the anterior neural fold.

In addition, given the unique content of the lamprey genome, which is extremely enriched with G and C nucleotides, we used special PCR buffer and Encyclo polymerase (a gift of the Evrogen company), which permits the effective amplification of such sequences.

**Figure 1:**
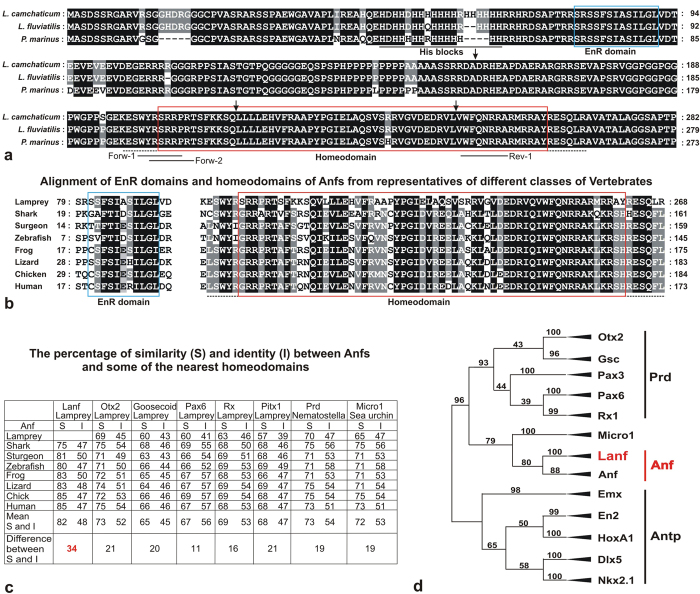
Sequences of lamprey *Anfs*. (**a**) Alignment of Lanfs from *L. camtschaticum, L. fluviatilis* and *P. marinus* (GenBank: KX245018, KX245019, KX245020). Homeodomains and the engrailed repressor domain (EnR) are indicated in red and blue frames, respectively; histidine-rich blocks are underlined with a solid black line; conservative motifs flanking the homeodomain are underlined with a dotted line; arrows indicate the positions of introns; the positions of the degenerate primers used for RT-PCR during *Lanf* cloning are indicated with solid lines. (**b**) Alignment of the EnR domains and homeodomains of *L. camtschaticum Lanf* with those of *Anfs* from different classes of vertebrates. (**c**) The percentages of similarity (S) and identity (I) between *Anfs* and some of the homeodomains of other types. The mean difference between the similarity (S) and identity (I) of the *Lanf* homeodomain with homeodomains of other *Anfs* (bottom line, highlighted by red) is much greater than the analogous values calculated for *Lanf* and other types of homeodomains (bottom line, in black). This fact indicates conservation of *Lanf* function during evolution. (**d**) Scheme of the neighbour-joining phylogenetic tree of *Anf* homeodomains and homeodomains of *Antp*- and *Prd*-class proteins (full trees constructed via the neighbour-joining and maximum likelihood methods are shown in Fig. S2, Fig. S3 and Fig. S4).

Subsequent RT-PCR performed based on this RNA sample with a set of *Anf*-specific degenerate oligos (see Materials and Methods), followed by cloning of the obtained fragments and their sequencing, yielded three clones from *L. camtschaticum* and two clones from *L. fluviatilis* containing the same insert, which showed closer homology to *Anf* sequences in other species than to any of the other known homeoboxes of lampreys. The remaining 5′ and 3′ fragments of this sequence were obtained via the suppression PCR-based Step-Out RACE technique^18^, which finally allowed us to establish the full coding sequences of the *L. camtschaticum* and *L. fluviatilis Anf* cDNAs and to clone them via RT-PCR with corresponding flanking primers. Importantly, with these cDNA sequences in hand, we were able to identify fragments of the genomic sequence of *Anf* among the *L. camtschaticum* genomic sequences deposited in GenBank. As a result, we established that similar to all known *Anfs* of other species, lamprey *Anf* is disrupted by three introns, two of which are within the homeobox (Fig. 1a, arrows).

To clone *Anf* from *P. marinus*, we used *Petromyzon* cDNA from the late neurula stage and flanking primers designed based on non-translated regions of the *L. camtschaticum Anf* mRNA.

The proteins encoded by the identified cDNAs exhibit all of the features characteristic of the *Anf* class (Fig. 1a). First, their homeodomains show a higher percentage of similarity with the homeodomains of *Anfs* than with any other type of homeodomain (Fig. 1b). Additionally, the *Lanf* homeodomain is flanked on both sides by specific short amino acid motifs that are peculiar to *Anf*-class proteins (Fig. 1a,b). Furthermore, like all other known *Anfs*, the *Lanfs* exhibit an engrailed-type repressor domain (EnR) in proximity to the N-terminus (Fig. 1a,b).

In addition, in contrast to the percentage of amino acid identity, the percentage of amino acid similarity between *Lanf* and homeodomains of the *Anf* class is much higher than between *Lanf* and other types of homeodomains, indicating that *Lanf* belongs to the *Anf* class. (Fig. 1c). Importantly, the phylogenetic relationships between lamprey *Anfs* correspond well to the known phylogenetic relationships between these species, with *P. marinus* being a more distant species from the two closely related species *L. camtschaticum* and *L. fluviatilis* (Fig. S2)^19^.

Finally, a close relationship of *Lanf* with *Anfs* from other classes of vertebrates was confirmed by their clustering in phylogenetic trees (Fig. 1d, Fig. S2, Fig. S3, Fig. S4).

In summary, it can be concluded that the identified homeoboxes are indeed lamprey *Anfs*.

### Analysis of the phylogenetic relationships of the *Anf* homeodomain indicates its possible hybrid origin from *Antp* and *Prd* classes

As can be observed from Fig. 1d, analysis of the phylogenetic relationships of *Anf* homeodomains in different classes of vertebrates, including the most basal species, the lampreys, with other known types of homeodomains revealed an intermediate position of *Anf* between the homeodomains of two large classes, *Antp* and *Prd*. This result led us to analyse the homology of different regions of the *Anf* homeodomain with the corresponding regions of the *Antp* and *Prd* homeodomains.

We found that the most of the *Anf* homeodomain, from its N-terminus (position 1) to the end of the protein sequence encoded by the 3rd exon (position 46), clustered with the homologous region of the *Prd* class of homeodomains via the neighbour-joining method (Fig. S1, Fig. S5). Moreover, this finding held true if only an N-fragment of this region (including the 1st alpha-helix) or its central fragment (including the 2nd helix) was used for clustering (not shown). In contrast, the C-terminal region of the *Anf* homeodomains from position 47 to 60 plus 4 amino acids of the conservative Anf homeodomain-flanking ESQ motif (positions 61–64), encoded by the last (4th) exon, were confidently grouped with *Antp*-class sequences (Fig. S1, Fig. S6). Thus, at least formally, the *Anf* homeodomain appears to be a hybrid of *Prd*- and *Antp*-class homeodomains.

It is also important to note that among all of the known invertebrate homeodomains, those showing the highest homology to *Anf* are the homeodomains of the Micro and Pmar proteins of sea urchins (Fig. 1d, Fig. S2, Fig. S3, Fig. S4). However, these proteins lack traits that are characteristic of *Anfs*, such as specific conservative sequences flanking the homeodomain, the EnR domain near the N-terminus and the location of the homeodomain near the C-terminus. Moreover, the exon-intron structure of their genes is different from that of *Anfs*. Therefore, one may conclude that *Anfs* appear to have no orthologues, at least in the genomes of modern invertebrates.

### Expression of *Lanf* in early embryogenesis

The temporal expression pattern of *Lanf* was investigated via qRT-PCR, and only very low expression of this gene was revealed before gastrulation. At the onset of gastrulation, its expression begins to increase slightly, but before the end of gastrulation returns to its pregastrulation level. Expression increases again, beginning from the late neurula stage (stage 19), reaches a maximum at the pharyngula stage (stage 22) and finally drops down to the background level by the hatching stage. Interestingly, in all other vertebrates, the expression of *Anf* gradually increases during gastrulation, reaching a maximum by the end of this stage, and then declines to the background level by the onset of the pharyngula stage (i.e., by the stage at which the expression of *Lanf* reaches its maximum in lamprey)^8^,^9^,^14^. Thus, obvious heterochrony is observed between *Lanf* expression in lamprey and the expression of its orthologues in other vertebrates.

**Figure 2:**
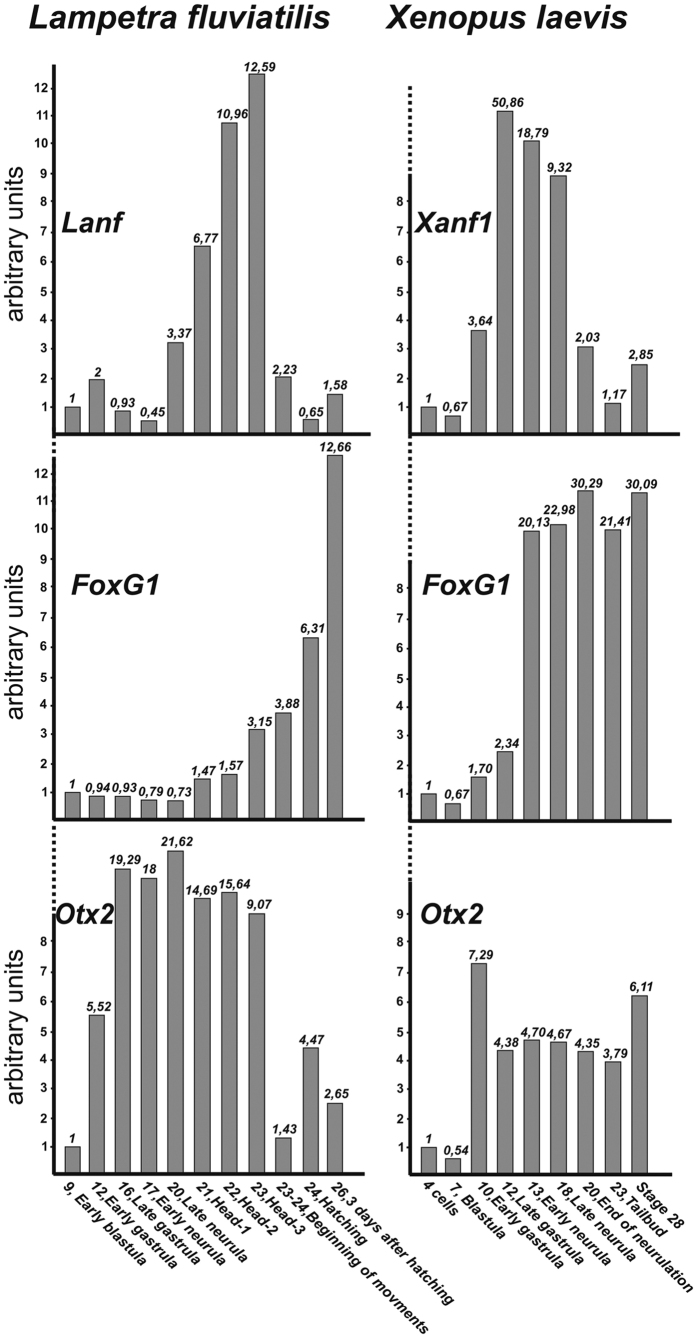
Comparison of the temporal expression pattern of *Lanf* with those of *FoxG1* and *Otx2* in lamprey (*L. fluviatilis*) and frog (*X. laevis*). qRT-PCR data obtained for *Anfs (Lanf* and *Xanf1*), *FoxG1* and *Otx2* in whole embryos collected at the stages indicated below were normalized relative to qRT-PCR data obtained for the housekeeping genes *EFalfa* and *ODC*. Stage numbers are indicated according to^17^,^34^. All data are from three experiments, average values are shown.

This heterochrony of *Lanf* expression is especially evident compared with the expression of another important anterior regulator, the homeobox gene *Otx2*, which in contrast to *Anf*, begins its expression at the same stage (i.e., the beginning of gastrulation) in the lamprey and *X. laevis* (Fig. 2). Interestingly, the telencephalic-specific marker gene *FoxG1*, whose expression is indirectly regulated by *Anf* in *X. laevis*^11^, begins to increase much later, after the end of neurulation, rather than the late gastrula stage in *X. laevis*. All of these findings indicate that in the lamprey, the telencephalon specification programme starts much later during embryogenesis than in other vertebrates.

**Figure 3:**
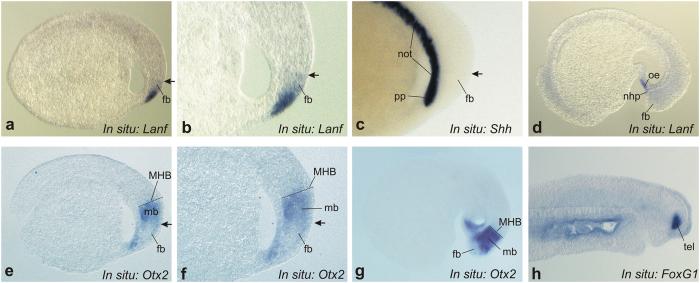
Analysis of the spatial expression pattern of *Lanf* in sagittal sections of *L. fluviatilis* embryos. (**a**–**c**) At stage 19, *Lanf* ((**a**) - overall view, (**b**) - enlarged view of anterior region) is expressed in the anterior neurectoderm, in the region corresponding to the stomodeal ectoderm (se) and the forebrain (fb), including the presumptive telencephalon and diencephalon. This region is located just above the region of *Shh* expression in the notochord (not) and the prechordal plate (pp) (**c**). An arrow indicates the dorsal limit of *Lanf* expression. Anterior is to the right; dorsal is at the top. (**d**) At stage 23, *Lanf* expression is observed only in the stomodeal ectoderm (se) and the nasohypophysial placode (nhp). (**е**,**f**) At the same stage shown for *Lanf* in (**a**,**b**), *Otx2* is expressed in a much broader area of the anterior neurectoderm, up to the presumptive mid-hindbrain boundary (MHB), and in the underlying mesoderm. Notably, the most anterior region of the neurectoderm, in which *Lanf* is expressed, is free of *Otx2* expression. (**g**) At stage 23, *Otx2* continues to be expressed in the anterior region, being inhibited in the most rostral part of it. (**h**) The expression of *FoxG1* marks the telencephalon beginning from stage 25 (see Materials and Methods).

The spatial expression of *Lanf* was studied via whole-mount *in situ* hybridization. Unfortunately, we were unable to detect expression via this method during gastrulation because of its very low level at this stage. At the same time, *Lanf* expression was observed beginning from stage 20 within the most anterior part of the brain anlage and in the surface ectoderm covering this brain area. As can be observed in Fig. 3a–c, the expression of *Lanf* in the neural tissue at this stage was localized just above the expression domain of *Shh* in the prechordal mesoderm, in the territory corresponding to the forebrain and, thus, to the presumptive telencephalon and rostral diencephalon^20^. This expression pattern is similar to the patterns of *Anf* expression in other studied vertebrate embryos^9^,^10^,^13^. As development proceeds, the expression of *Lanf* progressively ceases in the presumptive di- and telencephalic cells, persisting until stage 23 only in the oral ectoderm and the pituitary placode (Fig. 3d)^21^.

As observed in *X. laevis* embryos, *Anf i*s expressed within a subregion of the broader expression territory of *Otx2*, whose transcription is suppressed by *Anf* protein^11^. Thus, *Anf* “cleans” the most anterior part of the neuroectoderm of Otx2. As we have demonstrated, this function of *Anf* is critical for telencephalic development because it permits the telencephalic modulator *FoxG1* to be activated within this territory “cleaned” of Otx2.

Based on this assumption, we investigated the early expression patterns of the lamprey orthologues of *Otx2* and *FoxG1*in detail. Similar to *Otx2* expression in zebrafish, frog and mouse^11^,^22^,^23^, the expression of *Otx2* in lamprey was observed throughout the anterior part of the neural anlage, with a lower level of expression within the most rostral region, from which the telencephalon is derived. Importantly, this region of lower *Otx2* expression corresponds to the area of *Anf* expression, as in other species (Fig. 3e–g).

Unfortunately, we were unable to observe the expression of *FoxG1* at the early neurula stage. However, the expression of this gene at later stages indicates that, similar to *FoxG1* in other species, lamprey *FoxG1* is expressed within the region corresponding to the expression of *Lanf* (Fig. 3h).

### *Lanf* operates as a transcriptional suppressor

Our data reveal that the expression patterns of *Lanf, Otx2* and *FoxG1* are similar to those of these genes in other species. This finding indicates that *Lanf* may play a role similar to that of its orthologues in other species, i.e., permitting the expression of *FoxG1* owing to an inhibitory influence upon *Otx2* transcription. The presence of an engrailed-type repressor domain in *Lanf* (Fig. 1a and b) is also in agreement with the possibility that it functions as a transcriptional inhibitor.

To verify whether Lanf inhibits transcription, we tested its influence on the promoter of *Xanf1*, which is a target of its own protein product and, thus, presents a high probability of being a target of *Lanf* 24. To this end, we co-injected the *Xanf1* promoter-driven luciferase reporter mixed either with *Xanf1* or *Lanf* mRNA into *X. laevis* embryos at the 4-cell stage and analysed the luciferase signal at the midneurula stage. In both cases, we observed strong inhibition of the reporter compared with the control co-injected with the same reporter with EGFP mRNA (Fig. S7). These experiments confirmed the activity of *Lanf* as a transcriptional inhibitor.

### *Lanf* inhibits *Otx2* expression and promotes the expression of the telencephalon modulator *FoxG1*

**Figure 4:**
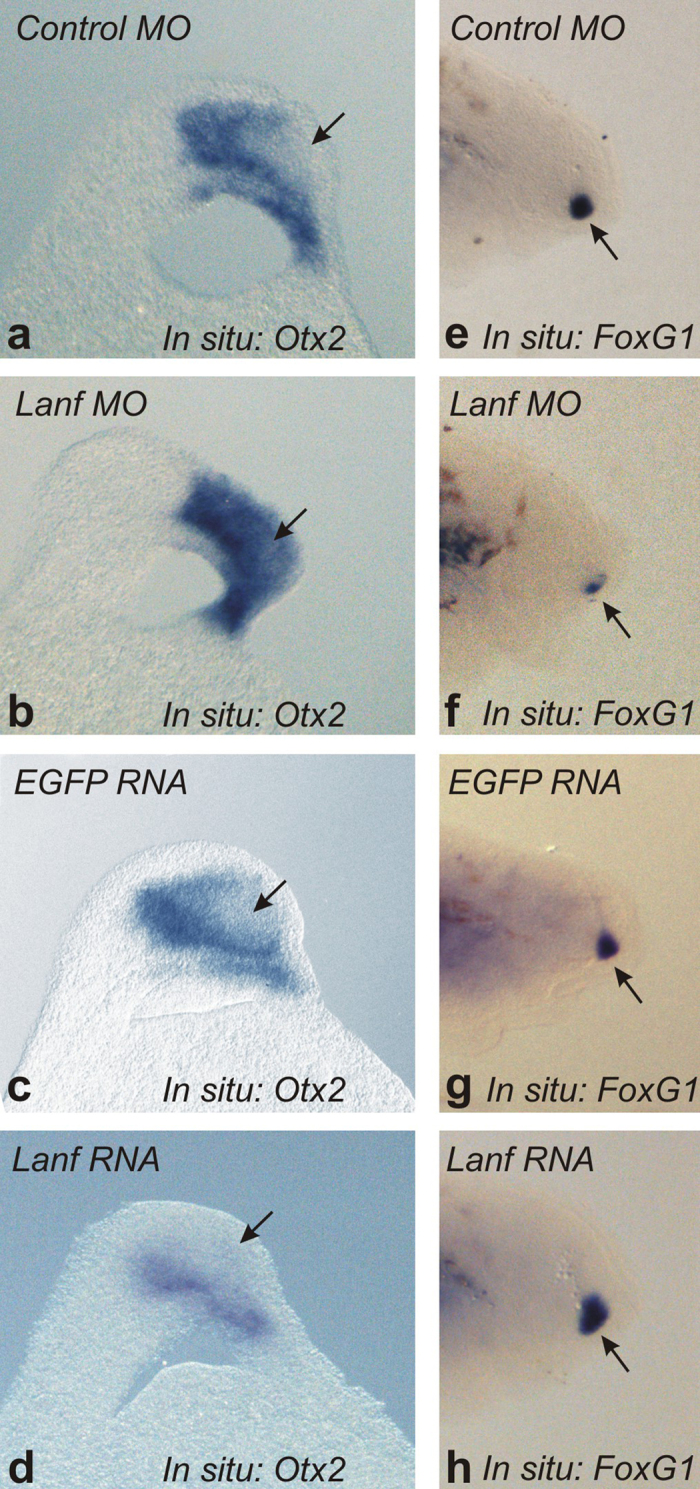
*Lanf* is responsible for the suppression of *Otx2* in the anterior neural plate and for the induction of *FoxG1* expression in this region. (**a–d**) In the control (**a**), the expression of *Otx2* is suppressed (arrow) in the anterior neural plate (100%, n = 35), but it is strongly activated (arrow) when *Lanf* is downregulated with an anti-sense MO targeting *Lanf* mRNA (70%, n = 50) (**b**) In contrast, it appears to be more broadly suppressed (arrow) if *Lanf* mRNA is overexpressed (76%, n = 55) (**d**) compared with the control EGFP mRNA overexpression (97%, n = 37). Anterior is at the top; dorsal is to the left. (**e–h**) An effect opposite that caused by *Lanf* upon *Otx2* expression is observed in the case of *FoxG1* expression (arrow) (100%, n = 30; 65%, n = 40; 95%, n = 42 and 70%, n = 45, respectively). Anterior is to the right; dorsal is at the top (see Materials and Methods).

As shown previously, down-regulation of *Anf* in frog embryos results in anterior expansion of *Otx2* domain, accompanied by a reduction of *FoxG1* expression, whereas overexpression of *Anf* elicits opposite effects^11^,^13^. To determine whether this result is also true for *Lanf*, we injected antisense morpholino oligonucleotides targeting *Lanf (Lanf* MO) or *Lanf* synthetic mRNA into lamprey embryos. As expected this expanded the *Otx2* domain anteriorly, towards the territory, in which its expression was weak in wild-type embryos (Fig. 4a and b). In turn, *Lanf* mRNA injection frequently reduced *Otx2* expression (Fig. 4c and d). In contrast, the opposite effect was observed for *FoxG1*, which is expressed in cells derived from the *Lanf* expression domain (Fig. 4e–h). Suppression of *Lanf* mRNA translation resulted in a reduction of *FoxG1* expression (Fig. 4e and f), while injection of *Lanf* mRNA elicited expansion of the *FoxG1* expression area (Fig. 4g and h). The observed expression abnormalities therefore confirmed an inhibitory influence of *Lanf* upon *Otx2* expression and its promotion of the expression of *FoxG1*.

## Discussion

**Figure 5:**
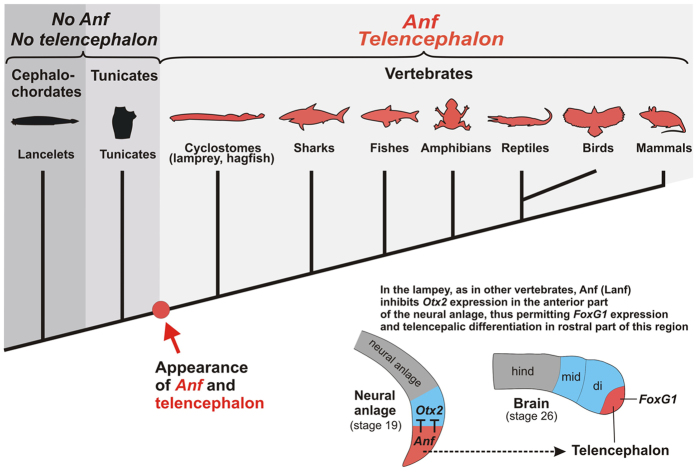
The appearance of the *Anf* homeobox class in ancestors of vertebrates elicited inhibition of *Otx2* in the rostral portion of the neural anlage, creating appropriate conditions for telencephalon emergence in this region.

In this work, we identified an *Anf*-class homeobox gene in lampreys and confirmed its essential role in lamprey telencephalic development, showing that *Anf* is indeed present not only in all classes of gnathostomes. but also in cyclostomes (Fig. 5). This finding confirms that the core mechanism responsible for the regionalization of the vertebrate brain arose before the divergence of cyclostomes and gnathostomes^25^. In turn, the lack of *Anf* in all invertebrates, including the nearest relatives of vertebrates, tunicates and cephalochordates, which have no anatomical structure homologous to the telencephalon, corroborates our previous hypothesis that the appearance of the *Anf* homeobox in vertebrate ancestors might be one of the events that provided appropriate conditions for the emergence of the telencephalon.

Interestingly, recent studies have demonstrated that in lancelets, the group of invertebrate chordates whose members exhibit the most similar body morphology to vertebrates, *FoxG1* is also expressed at the anterior end of the CNS, i.e., in the brain vesicle, which is thought to be homologous to the vertebrate diencephalon^26^. At first glance, this result is at odds with the hypothesis that the emergence of *Anf* in vertebrates was necessary for anterior *FoxG1* expression. However, expression of *FoxG1* can be observed in this region of the lancelet CNS only on the 3rd day of development, when the brain vesicle already appears to be well formed^26^. Moreover, *FoxG1* is expressed in this region in single scattered cells, whereas no expression of this gene is detected in the brain vesicle within a continuous territory, as observed in the case of *FoxG1* expression in the vertebrate telencephalic anlage, including that of the lamprey.

Notably, the expression of *FoxG1* and *Otx2* in mutually exclusive domains was recently reported in the embryos of *Saccoglossus kovalevskii*, a member of another invertebrate sister group of vertebrates, the hemichordates^27^. In embryos of these animals, *FoxG1* is expressed in scattered cells of the proboscis, the most anterior part of the *Saccoglossus* body, whereas *Otx2* expression is detected in the more posterior collar region. However, as no *Anf* was found in *Saccoglossus*, such mutual exclusion of *FoxG1* and *Otx2* expression is obviously ensured by other mechanisms.

Additionally, because telencephalic-like structures are not found in the *Saccoglossus* proboscis^6^, these results indicate that mutually exclusive expression of *FoxG1* and *Otx2* at the anterior end of the body is not sufficient by itself to ensure the development of the telencephalon. Importantly, as there is no firm evidence that extant hemichordates are the direct ancestors of vertebrates^6^, and as they don’t have telencephalon-like structures, this outcome does not contradict our hypothesis that in vertebrates, the mechanism responsible for *Otx2* down-regulation in cells of the presumptive telencephalon includes the expression of *Anf* as one of the necessary conditions.

Similar to other vertebrates, the expression of *Anf* in lamprey embryos is also observed in the adenohypophysis anlage. As in mouse embryos, the repressive activity of *Anf/Hesx1* is required at very early stages for adenohypophysis commitment^28^. Thus, our detection of *Anf* expression in the early adenohypophysis anlage is consistent with the previous suggestion that the programme responsible for this organ’s development was already present early in vertebrate evolution, before the divergence of cyclostomes and gnathostomates^21^,^25^.

Assuming that *Anf* is present only in vertebrates, it will be important to understand how this gene might have appeared during the evolution of vertebrates. As we have shown previously, the *Anf* homeodomain differs from other types of homeodomains in exhibiting an extremely high rate of amino acid substitutions^9^. Therefore, one possibility regarding the origin of *Anf* might be rapid evolution from a copy of some other homeobox gene that had undergone duplication in a vertebrate ancestor. Accordingly, early vertebrate evolution is known to have been accompanied by global genome rearrangements, including two rounds of whole-genome duplication^29^.

Our present analysis shows that the *Anf* homeodomain appears to be a hybrid of two different homeodomains, belonging to the Prd and Antp classes. Importantly, the N- and C-portions of Anf, which are the most like to the corresponding portions of the Prd and Antp homeodomains, respectively, are encoded by different exons, separated by a 3rd intron in the *Anf* genes in all species. Moreover, only these portions of the homeobox are separated by an intron in the majority of Prd-class genes and in some Antp-class genes, which is in agreement with the notion of a hybrid origin of the *Anf* homeodomain. Otherwise, it would be difficult to explain why the alleged connection of different genomic fragments did not result in mistakes in the homeodomain structure owing to a frame shift or the formation of a nonfunctional protein.

In addition, the hybrid hypothesis of the origin of *Anf* explains some features of its expression pattern and physiological functions. Indeed, if the 5′-region of the genomic sequence of *Anf* was derived from a *Prd*-class gene, then it may also have inherited the promoter region of this gene. Additionally, because many genes of the *Prd* class, such as *Gsc, Otx2, Pax6, Pitx*, and *Rx*, are characterized by expression in the anterior region of the embryo, this promoter inherited by *Anf* from the *Prd* gene may also govern its anterior expression. For instance, this promoter could come from a copy of some preliminarily duplicated gene of the Rx family, extant members of which show expression closely resembling that of *Anf*.

On the other hand, as shown by our work, a substantial portion of the *Anf* recognition helix, including four main residues (at positions 47, 50, 51 and 54) that undergo sequence-specific contacts with DNA, is probably inherited from one of the *Antp*-class genes. This group of genes is mainly expressed in the trunk region of embryos, and their protein products exert antagonistic effects on genes expressed rostrally. These traits of *Antp*-class proteins may explain the inhibitory influence of *Anf* on *Otx2* expression.

As we showed previously in *X. laevis* and have now confirmed in lamprey, *Anf* inhibits *Otx2* expression and thereby “cleans” Otx2 expression from the rostral region of the neural plate, which allows cells in the rostral neural plate to begin to express the telencephalic regulator *FoxG1*. When *Anf* is experimentally downregulated, *Otx2* expression expands to the presumptive telencephalic territory, which is accompanied by inhibition of *FoxG1* expression and posteriorisation of this territory^11^,^13^,^30^.

Accordingly, telencephalic specification appears to represent a peculiar “ground state” that can be reached by removal of Otx2, which could otherwise direct the presumptive telencephalic territory to a more posterior fate. At first glance, such a permissive strategy contradicts the fact that the telencephalon is the evolutionarily youngest brain unit because the “ground state” would intuitively be expected to be the oldest. To imagine how such an “inversion” could arise in evolution, it may be suggested that, as a first step, an inhibitory mechanism suppressing initial specification in a certain group of cells in the anterior region of the neural plate could have emerged. As we discussed above, this might have resulted from a genomic translocation that connected the 5′ region of a rostrally expressed *Prd* class gene, such as *Rx*, with the 3′ portion of a gene from the *Antp* class. Then, the progeny of this mutant ancestor, in which initial cell specification in the rostral region of the neural plate was inhibited, might obtain an advantage in natural selection because anterior cells become free to develop a new structure. Interestingly, the possibility that these ancient *Anf*-expressing cells could be stem-like cells with an uncertain fate was indirectly confirmed by the expression of *Anf* in mammalian ES cells, which are known as the cell type with the most uncertain fate^12^. Accordingly, we may speculate that the supposed translocation event resulting in the generation of *Anf*-expressing cells in the rostral neural plate could then have been co-opted in subsequent evolution as a necessary regulatory unit in the descendants’ developmental programme. Assuming this scenario to be true, the inhibition of *Otx2* by *Anf* that takes place in the embryos of all extant vertebrates might represent a trace of this ancient inhibitory mechanism that “cleaned out” the territory and was further used by natural selection as a ground state for the “construction” of the telencephalon.

Beginning from Haeckel’s conception of terminal addition, it is thought that evolutionary innovations may be more successfully accepted by natural selection if they appear in later stages of embryogenesis because they may result in less disturbance of the developmental programme in this case^31^,^32^. In this respect, the very early expression of *Anf* within the anterior neurectoderm (beginning from the midgastrula stage) of vertebrates appears to be contradictory to the fact that the telencephalon is the youngest brain unit. However, as shown herein, the expression of *Anf* in lampreys, the most basal extant vertebrate species, shows heterochrony with its expression in other species, as *Anf* expression begins within the neurectoderm of the lamprey embryo only at the late neurula stage. Additionally, the much later onset of the expression of the main telencephalic regulator, *FoxG1*, further confirms the later specification of the telencephalon in lamprey. Importantly, similar significant heterochrony in lamprey for another important gene regulating telencephalon specification, *Fgf8*, was reported previously^33^. All of these data are clearly in agreement with the fact that the telencephalon is the youngest brain unit, which could have emerged in vertebrate ancestors at considerably later stages of their embryonic development. This finding in turn suggests that changes occurred during subsequent evolution that pushed telencephalic specification to earlier stages of embryogenesis. Interestingly, this idea is reminiscent of terminal addition, according to which evolutionary innovations are added to the terminal stages of embryogenesis in ancestors and are then pushed into earlier embryonic stages of descendants.

## Materials and Methods

### Animals

All animal experiments were performed in accordance with guidelines approved by the Shemyakin-Ovchinnikov Institute of Bioorganic Chemistry (Moscow, Russia) Animal Committee and handled in accordance with the 1986 Animals (Scientific Procedures) Act and Helsinki Declaration.

*Lampetra fluviatilis, Lethenteron camtschaticum* and *Petromyzon marinus* adult lampreys were collected in the Saint Petersburg, Petropavlovsk-Kamchatski and Archangelsk districts, respectively. Embryos were obtained via artificial fertilization of eggs squeezed from pregnant females. The embryos were staged as described previously^17^. For *in situ* hybridization, embryos were fixed in MEMFA (3,7% formaldehyde, 100 mM MOPS, 2 mM EGTA, 1 mM MgSO_4_), dehydrated in methanol and kept at −20 °C. The *Xenopus laevis* embryos used for testing *Lanf* activity were obtained from laboratory frogs through artificial fertilization and staged according to the Nieuwkoop and Faber Normal Tables^34^.

### Cloning of *Lanf cDNA*

The homeobox-containing fragment of *Lanf* cDNA was obtained by using the Evrogen Kit for RT-PCR with degenerate oligos (see Supplementary). The positions of these oligos are shown in Fig. 1.

The homeobox sequence showing the highest homology to known Anfs from other vertebrates was chosen to design nested pairs of primers to obtain the 5′ and 3′ ends of *Lanf* cDNA via the Step-Out RACE method^18^. The cDNA fragment obtained through this method was cloned into the pGEM-T vector and sequenced. Finally, the cDNA sequence of Lanf was confirmed by obtaining the full-length cDNA with independent pairs of primers (see Supplementary) and sequencing several pGEM-T clones containing this cDNA.

### Cloning of other cDNAs for *in situ* hybridization

Fragments of the cDNAs of *FoxG1, Otx2, Shh* and *Lanf* to be used for *in situ* hybridization were obtained via RT-PCR with the primers shown in Supplementary.

### Bioinformatics

Phylogenetic analyses of protein sequences were performed via the neighbor-joining^35^ and maximum likehood^36^ methods using the MEGA6^37^ program. The percentage of replicate trees in which the associated taxa clustered together in the bootstrap test (1000 replicates) is shown next to the branches^38^. The trees were rooted with the UPGMA algorithm or by including the PouII homeobox as the outgroup sequence. Evolutionary distances were computed using the JTT matrix-based method^39^. Selection of species for analyses in the case of the OTX, Goosecoid, and *Anf* gene families was performed accordingly^40^. In other cases, the genes were taken from invertebrates (insect, nematode, sea urchin, sea anemone and ascidia) and vertebrates (lamprey, frog, mouse).

The homeodomain sequences and the names of the species included in the phylogenetic analyses are shown in Supplementary.

### RT-PCR and luciferase assays

For qRT-PCR, three groups of *L. camtschaticum* and *X. laevis* embryos were collected, obtaining 30 and 5 embryos, respectively, from each of the desired stages. Total RNA was extracted using an RNA isolation kit (MASHEREY-NAGEL) according to the manufacturer’s protocol. The concentration of the extracted RNA was measured with a Qubit® fluorometer (Invitrogen), while RNA integrity was checked visually via gel electrophoresis. The details of qRT PCR preparation and the reaction parameters and primers are shown in Supplementary.

The luciferase assay was performed as described in ref. 41 and Supplementary.

### Synthetic mRNA and morpholino

Synthetic *Lanf* and *Xanf1* mRNA was prepared with the mMessage Machine SP6 Kit (Ambion) after linearization of pCS2-based plasmids with NotI.

The following two variants of morpholino antisense oligonucleotides from Gene Tools (Fig. S8a) were injected at a final concentration of 0.4 mM in a volume of 3–4 nl.

1.   *Lanf MO1 (MO* corresponding to positions −20- + 5 of *L. fluviatilis* mRNA):

5′-GCCATCTCTCGAAAAGTAATTCACG;

2.   *Lanf MO2 (MO* corresponding to positions −46–22 of *L. fluviatilis* mRNA):

5′-ATTAGTTAATTGATCGGCGGTGGAA.

Importantly, *Lanf MO1* and *Lanf MO2* induced similar effects when they were injected into the embryos. A mismatched variant of *Lanf MO-1* was used as a negative control: *misLanf MO1*–5′- ACCAAGTCTCGTTAAGAAATTTGCG. The efficiency of the MOs was tested (see Supplementary).

All mRNA and MO were mixed with Fluorescein Lysine Dextran (FLD) (Invitrogen, 40 kD, 5 μg/μl) before injection.

### *In situ* hybridization

Whole-mount *in situ* hybridization was performed mainly as described previously^42^ with minor variations (see Supplementary).

Vibratome embryo sections with a thickness of 30 μm, hybridized in whole mounts and mounted in 4% agarose blocks, were prepared with a Microm HM 650 vibratome and photographed with a Leica M205 stereomicroscope.

## Additional Information

**How to cite this article**: Bayramov, A. V. *et al*. The presence of *Anf/Hesx1* homeobox gene in lampreys suggests that it could play an important role in emergence of telencephalon. *Sci. Rep.* **6**, 39849; doi: 10.1038/srep39849 (2016).

**Publisher's note:** Springer Nature remains neutral with regard to jurisdictional claims in published maps and institutional affiliations.

## Supplementary Material

Supplementary Dataset
