# Supplementary material for: The presence of Anf/Hesx1 homeobox gene in lampreys suggests that it could play an important role in emergence of telencephalon
Source: Sci Rep. 2016 Dec 23;6:39849. doi: 10.1038/srep39849 (PMC5180219; doi:10.1038/srep39849)
Supplement: Supplementary Dataset [file srep39849-s1.doc]

Supplementary Materials for

**The presence of *Anf/Hesx1* homeobox gene in lampreys suggests that it could play an important role in emergence of telencephalon**

Andrey V. Bayramov^1^, Galina V. Ermakova^1^, Fedor M. Eroshkin^1^, Alexandr V. Kucheryavyy^2^ , Natalia Y. Martynova^1^, Andrey G. Zaraisky^1*^

**These Supplementary Materials include:**

**Materials and Methods**

**Figure S1.**

**Figure S2.**

**Figure S3.**

**Figure S4.**

**Figure S5.**

**Figure S6.**

**Figure S7.**

**Figure S8.**

**Materials and Methods**

**Animals**

To obtain head protrusions at stage 20, embryonic envelopes were removed by forceps, head protrusions were excised by using microknife and glass stick and disintegrated in lysis buffer.

**Cloning of *Lanf* *cDNA***

Degenerated oligos for Lanf homeobox (see position of these oligos on **Fig. 1**):

Forward-1

5'-tggtatagXZgccgXZgZcc;

Forward-2

5'-agaZgccgXagZccXUgUac;

Reverse-1

5'-XgcacUXcgYttctgYaacca;

where U – A or C; X - A, G, C or T; Y- C or T; Z – A or G.

The first round of PCR (30 cycles) was done with Forward-1 and Reverse-1 primers. Then, the PCR mixture was diluted 1000 times and other 20 PCR cycles were performed by using Forward-2 and Reverse-1 primers. The resulting PCR product was cloned into pGEM-T vector and cDNA inserts of about 100 clones were sequenced.

Primers used for cloning the full coding sequence of *Lanf* cDNA:

*Lanf-Forw*: 5’-AGAACAAAATTCCACCGCCGA

*Lanf-Rev*: 5’- GTACAGGTGAGTGGGTGAGTCA

**Cloning of other cDNA for in situ hybridization**

Fragments of cDNA of *FoxG1*, *Otx2,* *Shh* and *Lanf* for in situ hybridization were obtained by RT-PCR with the following pairs of primers and cloned in *pGEM-T*:

*FoxG1-forw* GCCTCAACAAGTGCTTCGTGAAGGT

*FoxG1-rev* GTTATATACAGTTTGTATTTACAAGCCAT

*Otx2-forw* GCAGAGCGGCGGGCAGAGCAA 
*Otx2-rev* CCTCTCAGAGCACCTGGAACTT

*Lanf-forw* CGGCGCTCCAGAAGTTCATTCTC

*Lanf-rev* CACCGCGCGGAGCTGCGACTCG

*Shh-forw* GGAGCCAGCGGTCGCTACGA

*Shh-rev* TCCACGAGCACCGTGCCGCTC

The obtained PCR fragments were cloned into pGEM-T plasmid and sequenced.

**Bioinformatics**

Abbreviation of species names included in phylogenetic analysis:

Lc – lamprey *Lethenteron camtschaticum,* Lf – lamprey *Lampetra fluviatilis,* Pm – lamprey *Petromyzon marinus,* Cm – shark *Callorhinchus milii,* Ab – sturgeon *Acipenser baeri,* Dr – zebrafish *Danio rerio,* Xl – clawed frog *Xenopus laevis,* Ac – lizard *Anolis carolinensis,* Gg – chiken *Gallus gallus,* Mm - mouse *Mus musculus,* Hs – *Homo sapiens,* Hp – sea urchin *Hemicentrotus pulcherrimus,* Sp – sea urchin *Strongylocentrotus purpuratus,* Dm – fruit fly *Drosophila melanogaster,* Ce – nematode *Caenorhabditis elegans,* Nv – sea anemone *Nematostella vectensis,* Ci - ascidia *Ciona intestinalis*

Homeodomain sequences used for phylogenetic analyses shown on Fig1, FigS1, FigS2, FigS3 and FigS4 are below:

>LcAnf (*Lethenteron camtschaticum*, KX245018)

SRRPRTSFKKSQVLLLEHVFRAAPYPGIELAQSVSRRVGVDEDRVQVWFQNRRARMRRAY

>LfAnf (*Lampetra fluviatilis*, KX245019)

SRRPRTSFKKSQVLLLEHVFRAAPYPGIELAQSVSRRVGVDEDRVQVWFQNRRARMRRAY

>PmAnf (*Petromyzon marinus* KX245020)

SRRPRTSFKKSQVLLLEHVFRAAPYPGIELAQSVSHRVGVDEDRVQVWFQNRRARMRRAY

>CmAnf (*Callorhinchus milii*, XM_007890539.1)

GRRARTVFSRSQIEVLEEAFRRNCYPGIDVREQLAHKLTLDEDRIQIWFQNRRAKQKRSH

> AbAnf (*Acipenser baeri*, U65435)

GRRPRTAFSGTQIEVLESVFRVNPYPGIDVREELACKLELDEDRIQIWFQNRRAKLKRSH

> DrAnf (*Danio rerio*, NM_131349)

GRRPRTAFSSVQIKILESVFQVNSYPGIDIREELAKKLQLDEDRIQIWFQNRRAKLKRSH

>XlAnf (*Xenopus laevis*, X60099)

GRRPRTAFTRSQIEILENVFRVNSYPGIDVREELASKLALDEDRIQIWFQNRRAKLKRSH

>AcAnf (*Anolis carolinensis*, XM_003217755.3)

GRRPRTAFTRNQIEILESVFRVNSYPGIDVREELAQKLDLDEDRIQIWFQNRRAKLKRSH

>GgAnf (*Gallus gallus*, U65436.1)

GRRPRTAFTRNQIEVLENVFKMNSYPGIDIREELARKLDLEEDRIQIWFQNRRAKLKRSH

>HsAnf (*Homo sapiens*, U82811)

GRRPRTAFTQNQIEVLENVFRVNCYPGIDIREDLAQKLNLEEDRIQIWFQNRRAKLKRSH

>SpPmar1 (*Strongylocentrotus purpuratus*, NM_214508)

RRRRPTVFTELQLQILETAFNDNQYPDITAREQLASSLKIGEDRILVWFQNRRARLRRAS

>HpMicro1 (*Hemicentrotus pulcherrimus* , AB072733)

PRRRPTVFTELQLQILETAFNDNQYPGITTREQLASSLKLGEDRILVWFQNRRSRLRRAS

>XlOtx2 (*Xenopus laevis*, AAA85388)

QRRERTTFTRAQLDILEALFAKTRYPDIFMREEVALKINLPESRVQVWFKNRRAKCRQQQ

>HpOtxL (*Hemicentrotus pulcherrimus*, AB061768)

QRRERTTFTRAQLDVLETLFSRTRYPDIFMREEVAMKINLPESRVQVWFKNRRAKCRQQQ

# >SpOtx (*Strongylocentrotus purpuratus*, S76899)

# QRRERTTFTRAQLDVLETLFSRTRYPDIFMREEVAMKINLPESRVQVWFKNRRAKCRQQQ

# >DmOtd (*Drosophila melanogaster*, CAA41732)

QRRERTTFTRAQLDVLEALFGKTRYPDIFMREEVALKINLPESRVQVWFKNRRAKCRQQL

# >MmOTX (*Mus musculus*, NP_001273410.1)

QRRERTTFTRAQLDVLEALFAKTRYPDIFMREEVALKINLPESRVQVWFKNRRAKCRQQQ

# >NvOTX (*Nematostella vectensis*, ABB83750.1)

QRRERTTFTKNQLEILEELFAKTRYPDIFMREEVAIKINLPESRVQVWFKNRRAKARQQA

# >CiOtx (*Ciona intestinalis*, AAG59802.1)

QRRERTTFTRAQLDILEALFGKTRYPDIFMREEVALKINLPESRVQVWFKNRRAKCRQQV

# >LcOtxA (*Lethenteron camtschaticum*, BAA33409.1)

# QRRERTTFTRAQLDVLEALFSKTRYPDIFMREEVALKINLPESRVQVWFKNRRAKCRQQQ

>XlGoosecoid (*Xenopus laevis*, M63872)

KRRHRTIFTDEQLEALENLFQETKYPDVGTREQLARRVHLREEKVEVWFKNRRAKWRRQK

# >MmGsc **(*Mus musculus*,** EDL18791.1)

KRRHRTIFTDEQLEALENLFQETKYPDVGTREQLARKVHLREEKVEVWFKNRRAKWRRQK

# >SpGsc (*Strongylocentrotus purpuratus*, AF315231.1)

KRRHRTIFTEEQLEQLEATFEKTHYPDVMLREELAIKVDLKEERVEVWFKNRRAKWRKQK

# **>DmGsc** (*Drosophila****melanogaster*,** U52968.1)

KRRHRTIFTEEQLEQLEATFDKTHYPDVVLREQLALKVDLKEERVEVWFKNRRAKWRKQK

# >NvGsc (*Nematostella vectensis*, ABB72464.1)

KRRHRTIFTEEQLELLETTFQKTHYPDVLLREELAMKVDLKEERVEVWFKNRRAKWRKQK

# **> LcGsc (***Lethenteron camtschaticum*, AHE76143.1)

KRRHRTIFTDEQLGALEELFEQNQYPDVSMREQLARRVHLREERVEVWFKNRRAKWRRQK

>XenPax6 (*Xenopus laevis*, U77532)

LQRNRTSFTQEQIEALEKEFERTHYPDVFARERLAAKIDLPEARIQVWFSNRRAKWRREE

# >MmPax6 (***Mus musculus*,** NP_038655.1)

LQRNRTSFTQEQIEALEKEFERTHYPDVFARERLAAKIDLPEARIQVWFSNRRAKWRREE

# >LcPax6 (*Lethenteron camtschaticum*, BAB62531.1)

LQRNRTSFTQEQIEALEKEFERTHYPDVFARERLASKIDLPEARIQVWFSNRRAKWRREE

# > SpPax6 (*Strongylocentrotus purpuratus*, XP_003727316.1)

LQRNRTSFTAQQIEELEKEFERTHYPDVFARERLAQKIDLPEARIQVWFSNRRAKWRREE

# > DmEyeless (Drosophila melanogaster, NP_524628.2)

LQRNRTSFTNDQIDSLEKEFERTHYPDVFARERLAGKIGLPEARIQVWFSNRRAKWRREE

>XlPax3 (*Xenopus laevis*, AAI08574)

QRRGRTTFTAEQLEELERAFERTHYPDIYTREELAQRAKLTEARVQVWFSNRRARWRKQA

# >LcPax3 (*Lethenteron camtschaticum*, ADP37890.1)

QRRSRTTFTAEQLEELEKAFERTHYPDIYTREELAQRTKLTEARVQVWFSNRRARWRKQA

>XlRx1 (*Xenopus laevis*, BC170331.1)

HRRNRTTFTTYQLHELERAFEKSHYPDVYSREELAMKVNLPEVRVQVWFQNRRAKWRRQE

# >MmRx (***Mus musculus*,** NP_038861.2)

HRRNRTTFTTYQLHELERAFEKSHYPDVYSREELAGKVNLPEVRVQVWFQNRRAKWRRQE

# > DmDRx (*Drosophila melanogaster*, CAA11241.1)

HRRNRTTFTTYQLHELERAFEKSHYPDVYSREELAMKVNLPEVRVQVWFQNRRAKWRRQE

# > NvRX (*Nematostella vectensis*, ABB83751.1)

LRRNRTTFTTFQLHELERAFEKSHYPDVYTREELALKISLPEVRVQVWFQNRRAKWRRQE

# >SpSpp (*Strongylocentrotus purpuratus*, XP_782307.1)

HRRNRTTFTTYQLHELERAFEKSHYPDVYSREELALKVNLPEVRVQVWFQNRRAKWRRQE

>XlNkx2 (*Xenopus laevis*, AAG17405)

RRKRRVLFSQAQVYELERRFKQQKYLSAPEREHLASMIHLTPTQVKIWFQNHRYKMKRQA

# > LcNkx2.1 (*Lethenteron camtschaticum*, AMN92148.1)

RRKRRVLFSQAQVYELERRFKQQKYLSAPEREHLASMIHLTPTQVKIWFQNHRYKMKRQA

# > MmNkx-2.1 (***Mus musculus*,** NP_033411.3)

RRKRRVLFSQAQVYELERRFKQQKYLSAPEREHLASMIHLTPTQVKIWFQNHRYKMKRQA

# > SpNK2.1 (*Strongylocentrotus purpuratus*, NP_999800.1)

RRKRRVLFSQAQVYELERRFKQQKYLSAPEREHLANLINLTPTQVKIWFQNHRYKMKRQT

# > CeNK2 (*Caenorhabditis elegans*, AAB81844.1)

RRKRRVLFSQAQVYELERRFKQAKYLTAPEREQLANSIRLTPTQVKIWFQNHRYKCKRQE

# > NvNK2-Tinman (*Nematostella vectensis*, ABG67775.1)

RRKPRVLFSQAQVYELERRFKGQKYLSAPERDHLASLLKLTPNQVKIWFQNKRYKCKKQA

> DmScarecrow (*Drosophila melanogaster*,  AAF26436.1)

RRKRRVLFTQAQVYELERRFKQQRYLSAPEREHLASLIHLTPTQVKIWFQNHRYKCKRQA

>XlDlx5 (*Xenopus laevis*, NM_001090564.1)

IRKPRTIYSSFQLAALQRRFQKTQYLALPERAELAASLGLTQTQVKIWFQNKRSKIKKIM

> LcDlx (*Lethenteron camtschaticum*, BAH03341.1)

IRKPRTIYSSFQLAALQRRFQQTQYLALPERAELAASLGVTQTQVKIWFQNRRSKFKKIG

# > MmDLX5 (***Mus musculus*,** NP_034186.2)

VRKPRTIYSSFQLAALQRRFQKTQYLALPERAELAASLGLTQTQVKIWFQNKRSKIKKIM

> DmDll (*Drosophila melanogaster*, AAB24059.1)

MRKPRTIYSSLQLQQLNRRFQRTQYLALPERAELAASLGLTQTQVKIWFQNRRSKYKKMM

> SpDll (*Strongylocentrotus purpuratus*, NP_001123282.1)

LRKPRTIYTSLQLQQLNQRFHQTQYLALPERAELAASLGLTQTQVKIWFQNRRSKYKKIL

> NvDLX (*Nematostella vectensis*, ABB86447.1)

IRKPRTIYSSFQLRELNKRFIKTQYLALPERADLAAYLGLTQTQVKIWFQNRRSKFKKTL

>XlEmx1 (*Xenopus laevis*, NM_001093430)

PKRIRTAFSPSQLLRLERSFEKNHYVVGAERKQLATGLSLSETQVKVWFQNRRTKYKRQK

# > LcEmx (*Lethenteron camtschaticum*, BAB13506.1)

PKRIRTAFSPSQLLRLEHAFEKNHYVVGAERKQLASSLSLSETQVKVWFQNRRTKYKRQK

> MmEmx1 (*Mus musculus*, CAA48752.1)

PKRIRTAFSPSQLLRLERAFEKNHYVVGAERKQLAGSLSLSETQVKVWFQNRRTKYKRQK

# > DmEmpty spiracles (*Drosophila melanogaster*, NP_731868.1)

PKRIRTAFSPSQLLKLEHAFESNQYVVGAERKALAQNLNLSETQVKVWFQNRRTKHKRMQ

> SpEmx1pred (*Strongylocentrotus purpuratus*, XP_783008.1)

PKRIRTAFSPSQLLRLENAFEKNHYVVGAERKQLAASLNLTETQVKVWFQNRRTKYKRIK

# > NvEMXa (*Nematostella vectensis*, ABB86466.1)

PKRIRTAFTPTQLLHLENAFEKNHYIVGTERKQLASYLNLSETQIKVWFQNRRTKWKRQQ

>XlEn2 (*Xenopus laevis*, NM_001101743)

DKRPRTAFTADQLQRLKAEFQTNRYLTEQRRQSLAQELSLNESQIKIWFQNKRAKIKKAT

# > LcEngrailedA (*Lethenteron camtschaticum*, ABU41241.1)

EKRPRTAFSSEQLSRLKAEFQASRYLTEARRQALAQELQLNEAQIKIWFQNKRAKLKKAS

# > MmEn2 (***Mus musculus*,** CAA68362.1)

DKRPRTAFTAEQLQRLKAEFQTNRYLTEQRRQSLAQELSLNESQIKIWFQNKRAKIKKAT

# > DmEngrailed (*Drosophila melanogaster*, BAN82731.1)

EKRPRTAFSSEQLARLKREFNENRYLTERRRQQLSSELGLNEAQIKIWFQNKRAKIKKST

# > SpEngr-like pred (*Strongylocentrotus purpuratus*, XP_794753.1)

EKRPRTAFSASQLQRLKQEFQQSNYLTEQRRRALAKELTLSESQIKIWFQNKRAKIKKAT

>XlHoxA1 (*Xenopus laevis*, NP_001079188)

PNTARTNFTTKQLTELEKEFHFNKYLTRARRVEIAAALQLNETQVKIWFQNRRMKQKKRE

# **>** LcHox1 (Lethenteron camtschaticum, BAF63519.1)

IATQRTNFSTKQLTELEKEFHFNKYLTRARRVEIAAALQLNETQVKIWFQNRRMKQKKRE

# > MmHoxA1 (*Mus musculus*, NP_034579.3)

PNAVRTNFTTKQLTELEKEFHFNKYLTRARRVEIAASLQLNETQVKIWFQNRRMKQKKRE

# > SpHox-B1pred (*Strongylocentrotus purpuratus*, XP_781966.2)

NNNGRTNFTNKQLTELEKEFHFNKYLTRARRIEIAAMLGLNETQVKIWFQNRRMKEKKKM

# > Pou2 (*Xenopus laevis*, CAA41781.1)

KRKKRTSIEVSVKGVLETHFLKCPKPAALEITSLADSLQLEKEVVRVWFCNRRQKEKRMT

Sequences of N-part of homeodomain used for phylogenetic analysis shown on FigS5 are below:

>LcAnf (*Lethenteron camtschaticum*, KX245018)

SRRPRTSFKKSQVLLLEHVFRAAPYPGIELAQSVSRRVGVDEDRVQ

>LfAnf (*Lampetra fluviatilis*, KX245019)

SRRPRTSFKKSQVLLLEHVFRAAPYPGIELAQSVSRRVGVDEDRVQ

>PmAnf (*Petromyzon marinus* KX245020)

SRRPRTSFKKSQVLLLEHVFRAAPYPGIELAQSVSHRVGVDEDRVQ

>CmAnf (*Callorhinchus milii*, XM_007890539.1)

GRRARTVFSRSQIEVLEEAFRRNCYPGIDVREQLAHKLTLDEDRIQ

> AbAnf (*Acipenser baeri*, U65435)

GRRPRTAFSGTQIEVLESVFRVNPYPGIDVREELACKLELDEDRIQ

> DrAnf (*Danio rerio*, NM_131349)

GRRPRTAFSSVQIKILESVFQVNSYPGIDIREELAKKLQLDEDRIQ

>XlAnf (*Xenopus laevis*, X60099)

GRRPRTAFTRSQIEILENVFRVNSYPGIDVREELASKLALDEDRIQ

>AcAnf (*Anolis carolinensis*, XM_003217755.3)

GRRPRTAFTRNQIEILESVFRVNSYPGIDVREELAQKLDLDEDRIQ

>GgAnf (*Gallus gallus*, U65436.1)

GRRPRTAFTRNQIEVLENVFKMNSYPGIDIREELARKLDLEEDRIQ

>HsAnf (*Homo sapiens*, U82811)

GRRPRTAFTQNQIEVLENVFRVNCYPGIDIREDLAQKLNLEEDRIQ

>XlOtx2 (*Xenopus laevis*, AAA85388)

QRRERTTFTRAQLDILEALFAKTRYPDIFMREEVALKINLPESRVQ

>HpOtxL (*Hemicentrotus pulcherrimus*, AB061768)

QRRERTTFTRAQLDVLETLFSRTRYPDIFMREEVAMKINLPESRVQ

# >SpOtx (*Strongylocentrotus purpuratus*, S76899)

# QRRERTTFTRAQLDVLETLFSRTRYPDIFMREEVAMKINLPESRVQ

# >DmOtd (*Drosophila melanogaster*, CAA41732)

QRRERTTFTRAQLDVLEALFGKTRYPDIFMREEVALKINLPESRVQ

# >MmOTX (*Mus musculus*, NP_001273410.1)

QRRERTTFTRAQLDVLEALFAKTRYPDIFMREEVALKINLPESRVQ

# >NvOTX (*Nematostella vectensis*, ABB83750.1)

QRRERTTFTKNQLEILEELFAKTRYPDIFMREEVAIKINLPESRVQ

# >CiOtx (*Ciona intestinalis*, AAG59802.1)

QRRERTTFTRAQLDILEALFGKTRYPDIFMREEVALKINLPESRVQ

# >LcOtxA (*Lethenteron camtschaticum*, BAA33409.1)

QRRERTTFTRAQLDVLEALFSKTRYPDIFMREEVALKINLPESRVQ

>XlGoosecoid (*Xenopus laevis*, M63872)

KRRHRTIFTDEQLEALENLFQETKYPDVGTREQLARRVHLREEKVE

# >MmGsc **(*Mus musculus*,** EDL18791.1)

KRRHRTIFTDEQLEALENLFQETKYPDVGTREQLARKVHLREEKVE

# >SpGsc (*Strongylocentrotus purpuratus*, AF315231.1)

KRRHRTIFTEEQLEQLEATFEKTHYPDVMLREELAIKVDLKEERVE

# **>DmGsc** (*Drosophila****melanogaster*,** U52968.1)

KRRHRTIFTEEQLEQLEATFDKTHYPDVVLREQLALKVDLKEERVE

# >NvGsc (*Nematostella vectensis*, ABB72464.1)

KRRHRTIFTEEQLELLETTFQKTHYPDVLLREELAMKVDLKEERVE

# **> LcGsc (***Lethenteron camtschaticum*, AHE76143.1)

KRRHRTIFTDEQLGALEELFEQNQYPDVSMREQLARRVHLREERVE

>XenPax6 (*Xenopus laevis*, U77532)

LQRNRTSFTQEQIEALEKEFERTHYPDVFARERLAAKIDLPEARIQ

# >MmPax6 (***Mus musculus*,** NP_038655.1)

LQRNRTSFTQEQIEALEKEFERTHYPDVFARERLAAKIDLPEARIQ

# >LcPax6 (*Lethenteron camtschaticum*, BAB62531.1)

LQRNRTSFTQEQIEALEKEFERTHYPDVFARERLASKIDLPEARIQ

# > SpPax6 (*Strongylocentrotus purpuratus*, XP_003727316.1)

LQRNRTSFTAQQIEELEKEFERTHYPDVFARERLAQKIDLPEARIQ

# > DmEyeless (*Drosophila melanogaster*, NP_524628.2)

LQRNRTSFTNDQIDSLEKEFERTHYPDVFARERLAGKIGLPEARIQ

>XlPax3 (*Xenopus laevis*, AAI08574)

QRRGRTTFTAEQLEELERAFERTHYPDIYTREELAQRAKLTEARVQ

# >LcPax3 (*Lethenteron camtschaticum*, ADP37890.1)

QRRSRTTFTAEQLEELEKAFERTHYPDIYTREELAQRTKLTEARVQ

>XlRx1 (*Xenopus laevis*, BC170331.1)

HRRNRTTFTTYQLHELERAFEKSHYPDVYSREELAMKVNLPEVRVQ

# >MmRx (***Mus musculus*,** NP_038861.2)

HRRNRTTFTTYQLHELERAFEKSHYPDVYSREELAGKVNLPEVRVQ

# > DmDRx (*Drosophila melanogaster*, CAA11241.1)

HRRNRTTFTTYQLHELERAFEKSHYPDVYSREELAMKVNLPEVRVQ

# > NvRX (*Nematostella vectensis*, ABB83751.1)

LRRNRTTFTTFQLHELERAFEKSHYPDVYTREELALKISLPEVRVQ

# >SpSpp (*Strongylocentrotus purpuratus*, XP_782307.1)

HRRNRTTFTTYQLHELERAFEKSHYPDVYSREELALKVNLPEVRVQ

>XlNkx2 (*Xenopus laevis*, AAG17405)

RRKRRVLFSQAQVYELERRFKQQKYLSAPEREHLASMIHLTPTQVK

# > NKX2.1 (*Lethenteron camtschaticum*, AMN92148.1)

RRKRRVLFSQAQVYELERRFKQQKYLSAPEREHLASMIHLTPTQVK

# > MmNkx-2.1 (***Mus musculus*,** NP_033411.3)

RRKRRVLFSQAQVYELERRFKQQKYLSAPEREHLASMIHLTPTQVK

# > SpNK2.1 (*Strongylocentrotus purpuratus*, NP_999800.1)

RRKRRVLFSQAQVYELERRFKQQKYLSAPEREHLANLINLTPTQVK

# > CeNK2 (*Caenorhabditis elegans*, AAB81844.1)

RRKRRVLFSQAQVYELERRFKQAKYLTAPEREQLANSIRLTPTQVK

# > NvNK2-Tinman (*Nematostella vectensis*, ABG67775.1)

RRKPRVLFSQAQVYELERRFKGQKYLSAPERDHLASLLKLTPNQVK

> DmScarecrow (*Drosophila melanogaster*,  AAF26436.1)

RRKRRVLFTQAQVYELERRFKQQRYLSAPEREHLASLIHLTPTQVK

>XlDlx5 (*Xenopus laevis*, NM_001090564.1)

IRKPRTIYSSFQLAALQRRFQKTQYLALPERAELAASLGLTQTQVK

> LcDlx (*Lethenteron camtschaticum*, BAH03341.1)

IRKPRTIYSSFQLAALQRRFQQTQYLALPERAELAASLGVTQTQVK

# > MmDLX5 (***Mus musculus*,** NP_034186.2)

VRKPRTIYSSFQLAALQRRFQKTQYLALPERAELAASLGLTQTQVK

> DmDll (*Drosophila melanogaster*, AAB24059.1)

MRKPRTIYSSLQLQQLNRRFQRTQYLALPERAELAASLGLTQTQVK

> SpDll (*Strongylocentrotus purpuratus*, NP_001123282.1)

LRKPRTIYTSLQLQQLNQRFHQTQYLALPERAELAASLGLTQTQVK

> NvDLX (*Nematostella vectensis*, ABB86447.1)

IRKPRTIYSSFQLRELNKRFIKTQYLALPERADLAAYLGLTQTQVK

>XlEmx1 (*Xenopus laevis*, NM_001093430)

PKRIRTAFSPSQLLRLERSFEKNHYVVGAERKQLATGLSLSETQVK

# > LcEMX (*Lethenteron camtschaticum*, BAB13506.1)

PKRIRTAFSPSQLLRLEHAFEKNHYVVGAERKQLASSLSLSETQVK

> MmEmx1 (*Mus musculus*, CAA48752.1)

PKRIRTAFSPSQLLRLERAFEKNHYVVGAERKQLAGSLSLSETQVK

# > DmEmpty spiracles (*Drosophila melanogaster*, NP_731868.1)

PKRIRTAFSPSQLLKLEHAFESNQYVVGAERKALAQNLNLSETQVK

> SpEMX1pred (*Strongylocentrotus purpuratus*, XP_783008.1)

PKRIRTAFSPSQLLRLENAFEKNHYVVGAERKQLAASLNLTETQVK

# > NvEMXa (*Nematostella vectensis*, ABB86466.1)

PKRIRTAFTPTQLLHLENAFEKNHYIVGTERKQLASYLNLSETQIK

>XlEn2 (*Xenopus laevis*, NM_001101743)

DKRPRTAFTADQLQRLKAEFQTNRYLTEQRRQSLAQELSLNESQIK

# > LcEngrailedA (*Lethenteron camtschaticum*, ABU41241.1)

EKRPRTAFSSEQLSRLKAEFQASRYLTEARRQALAQELQLNEAQIK

# > MmEn2 (***Mus musculus*,** CAA68362.1)

DKRPRTAFTAEQLQRLKAEFQTNRYLTEQRRQSLAQELSLNESQIK

# > DmEngrailed (*Drosophila melanogaster*, BAN82731.1)

EKRPRTAFSSEQLARLKREFNENRYLTERRRQQLSSELGLNEAQIK

# > SpEngr-like pred (*Strongylocentrotus purpuratus*, XP_794753.1)

EKRPRTAFSASQLQRLKQEFQQSNYLTEQRRRALAKELTLSESQIK

>XlHoxA1 (*Xenopus laevis*, NP_001079188)

PNTARTNFTTKQLTELEKEFHFNKYLTRARRVEIAAALQLNETQVK

# **>** LcHox1 (*Lethenteron camtschaticum*, BAF63519.1)

IATQRTNFSTKQLTELEKEFHFNKYLTRARRVEIAAALQLNETQVK

# > MmHoxA1 (*Mus musculus*, NP_034579.3)

PNAVRTNFTTKQLTELEKEFHFNKYLTRARRVEIAASLQLNETQVK

# > SpHox-B1pred (*Strongylocentrotus purpuratus*, XP_781966.2)

NNNGRTNFTNKQLTELEKEFHFNKYLTRARRIEIAAMLGLNETQVK

Sequences of C-part of homeodomain used for phylogenetic analysis shown on FigS6 are below:

>LcAnf (*Lethenteron camtschaticum*, KX245018)

VWFQNRRARMRRAYRESQ

>LfAnf (*Lampetra fluviatilis*, KX245019)

VWFQNRRARMRRAYRESQ

>PmAnf (*Petromyzon marinus* KX245020)

VWFQNRRARMRRAYRESQ

>CmAnf (*Callorhinchus milii*, XM_007890539.1)

IWFQNRRAKQKRSHHESQ

> AbAnf (*Acipenser baeri*, U65435)

IWFQNRRAKLKRSHRESQ

> DrAnf (*Danio rerio*, NM_131349)

IWFQNRRAKLKRSHRESQ

>XlAnf (*Xenopus laevis*, X60099)

IWFQNRRAKLKRSHRESQ

>AcAnf (*Anolis carolinensis*, XM_003217755.3)

IWFQNRRAKLKRSHRESQ

>GgAnf (*Gallus gallus*, U65436.1)

IWFQNRRAKLKRSHRESQ

>HsAnf (*Homo sapience*, U82811)

IWFQNRRAKLKRSHRESQ

>XlOtx2 (*Xenopus laevis*, AAA85388.1)

VWFKNRRAKCRQQQQQQQ

>HpOtxL (*Hemicentrotus pulcherrimus*, AB061768)

VWFKNRRAKCRQQQQQQQ

# >SpOtx (*Strongylocentrotus purpuratus*, S76899)

# VWFKNRRAKCRQQQQQQQ

# >DmOtd (*Drosophila melanogaster*, CAA41732)

VWFKNRRAKCRQQLQQQQ

# >MmOTX (*Mus musculus*, NP_001273410.1)

VWFKNRRAKCRQQQQQQQ

# >CiOtx (*Ciona intestinalis*, AAG59802.1)

VWFKNRRAKCRQQVQQQQ

# >LcOtxA (*Lethenteron camtschaticum*, BAA33409.1)

VWFKNRRAKCRQQQQSGG

>XlGoosecoid (*Xenopus laevis*, M63872)

VWFKNRRAKWRRQKRSSS

# >MmGsc **(*Mus musculus*,** EDL18791.1)

VWFKNRRAKWRRQKRSSS

# >SpGsc (*Strongylocentrotus purpuratus*, AF315231.1)

VWFKNRRAKWRKQKREQQ

# **>DmGsc** (*Drosophila****melanogaster*,** U52968.1)

VWFKNRRAKWRKQKREEQ

# **> LcGsc (***Lethenteron camtschaticum*, AHE76143.1)

VWFKNRRAKWRRQKHAAT

>XenPax6 (*Xenopus laevis*, U77532)

VWFSNRRAKWRREEKLRN

# >MmPax6 (***Mus musculus*,** NP_038655.1)

VWFSNRRAKWRREEKLRN

# >LcPax6 (*Lethenteron camtschaticum*, BAB62531.1)

VWFSNRRAKWRREEKLRN

# > SpPax6 (*Strongylocentrotus purpuratus*, XP_003727316.1)

VWFSNRRAKWRREEKLRN

# > DmEyeless (*Drosophila melanogaster*, NP_524628.2)

VWFSNRRAKWRREEKLRN

>XlPax3 (*Xenopus laevis*, AAI08574)

VWFSNRRARWRKQAGANQ

# >LcPax3 (*Lethenteron camtschaticum*, ADP37890.1)

VWFSNRRARWRKQAGANQ

>XlRx1 (*Xenopus laevis*, BC170331.1)

VWFQNRRAKWRRQEKLEV

# >MmRx (***Mus musculus*,** NP_038861.2)

VWFQNRRAKWRRQEKLEV

# > DmDRx (*Drosophila melanogaster*, CAA11241.1)

VWFQNRRAKWRRQEKSES

# >SpSpp (*Strongylocentrotus purpuratus*, XP_782307.1)

VWFQNRRAKWRRQEKMEA

>XlNkx2 (*Xenopus laevis*, AAG17405)

IWFQNHRYKMKRQAKDKA

# > NKX2.1 (*Lethenteron camtschaticum*, AMN92148.1)

IWFQNHRYKMKRQAKDKA

# > MmNkx-2.1 (***Mus musculus*,** NP_033411.3)

IWFQNHRYKMKRQAKDKA

# > SpNK2.1 (*Strongylocentrotus purpuratus*, NP_999800.1)

IWFQNHRYKMKRQTKEKN

# > CeNK2 (*Caenorhabditis elegans*, AAB81844.1)

IWFQNHRYKCKRQEKEKA

> DmScarecrow (*Drosophila melanogaster*,  AAF26436.1)

IWFQNHRYKCKRQAKRKA

>XlDlx5 (*Xenopus laevis*, NM_001090564.1)

IWFQNKRSKIKKIMKNGE

> LcDlx (*Lethenteron camtschaticum*, BAH03341.1)

IWFQNRRSKFKKIGKHG

# > MmDLX5 (***Mus musculus*,** NP_034186.2)

IWFQNKRSKIKKIMKNGE

> DmDll (*Drosophila melanogaster*, AAB24059.1)

IWFQNRRSKYKKMMKAAQ

> SpDll (*Strongylocentrotus purpuratus*, NP_001123282.1)

IWFQNRRSKYKKILKQQN

>XlEmx1 (*Xenopus laevis*, NM_001093430)

VWFQNRRTKYKRQKLEEE

# > LcEMX (*Lethenteron camtschaticum*, BAB13506.1)

VWFQNRRTKYKRQKLEEE

> MmEmx1 (*Mus musculus*, NP_034261.1)

VWFQNRRTKYKRQKLEEE

# > DmEmpty spiracles (*Drosophila melanogaster*, NP_731868.1)

VWFQNRRTKHKRMQQEDE

> SpEMX1pred (*Strongylocentrotus purpuratus*, XP_783008.1)

VWFQNRRTKYKRIKSEEE

>XlEn2 (*Xenopus laevis*, NM_001101743)

IWFQNKRAKIKKATGNKN

# > LcEngrailedA (*Lethenteron camtschaticum*, ABU41241.1)

IWFQNKRAKLKKASGVRN

# > MmEn2 (***Mus musculus*,** CAA68362.1)

IWFQNKRAKIKKATGNKN

# > DmEngrailed (*Drosophila melanogaster*, BAN82731.1)

IWFQNKRAKIKKSTGSKN

# > SpEngr-like pred (*Strongylocentrotus purpuratus*, XP_794753.1)

IWFQNKRAKIKKATGLKN

>XlHoxA1 (*Xenopus laevis*, NP_001079188)

IWFQNRRMKQKKREKEGH

# **>** LcHox1 (*Lethenteron camtschaticum*, BAF63519.1)

IWFQNRRMKQKKREKEGH

# > MmHoxA1 (*Mus musculus*, NP_034579.3)

IWFQNRRMKQKKREKEGL

# > SpHox-B1pred (*Strongylocentrotus purpuratus*, XP_781966.2)

IWFQNRRMKEKKKMKECI

**RT-PCR and luciferase assay.**

For the first strand synthesis 250 ng of total RNA extracted from each sample was reverse transcribed in 20 μl of final volume by M-MLV reverse transcriptase (Promega) in presence of 10 pmol of oligo-dT primer (Evrogen), according to the manufacturer's guidelines (Promega) (+RT sample). In parallel, same reaction was assembled in each case without adding of M-MLV reverse transcriptase (-RT control). For qPCR reaction, which was performed on DT*Prim4* (DNA Technology), 2μl of +RT and -RT solutions of each type were mixed in parallel tubes with qPCRmix-HS SYBR (x5, Evrogen), corresponding primers (5pmol each) and milli-Q water till the final volume 25 μl. A standard 40-cycle program with hot start was used; the annealing temperature was 59°C, elongation – 72°C and melting 95 °C, all lasted for 25 seconds. The PCR data were imported into Microsoft Excel and analyzed by using the ΔΔ**C**_t_ method. The geometric mean of expression of two reference housekeeping genes: *ornithine decarboxylase* (ODC) and *elongation factor 1alpa* (*EF1alpha*) was used for normalization of the target genes expression levels. The following pairs of primers designed on the base o sequences deposited in GenBank were used:

*L. camtschaticum:*

*FoxG1-forw* CTTTCGGGACTTACCGTTCCA and

*FoxG1-rev* CCACTTGACTTTGCTGCTGA;

*Lanf-forw* CGCACGTCCTTCAAGAAGTCG and

*Lanf-rev* GACCCCCCAGCGAGAGCCGTCG;

*Otx2-forw* GCAGAGCGGCGGGCAGAGCAA  and

*Otx2-rev* CCTCTCAGAGCACCTGGAACTT;

*EF1alfa-forw* AGAACGTGTCTGTCAAGGATGT and

*EF1alfa-rev* TAGCCGGCATTGATCTGGCCA;

*ODC-forw* CCGTCGGTATCATCGCCAAG and

*ODC-rev* CGAAGAGGATGCAGTTGAAG;

*X. laevis*:

*OTX2a-forw* TTCAATGCTGACTGCTTGGAT and

*OTX2a-rev* AGATGAGGTTTGGCCCGAG;

*FoxG1-forw* AACAAGCAGGGCTGGCAGAA and

*FoxG1-rev* CCGCTCTATCCATAAAGGTG;

*Xanf1-forw* CCGCAGAAGAGGAGACAAAG and

*Xanf1-rev*TAGTGAAAGCAGTTCGGGGT;

*EF-forw* TCATACAGCTCATATTGCTTGTAAGT and

*EF-rev* CAAGTGGAGGATAGTCTGAGAA;

*ODC-forw* GCCATTGTGAAGACTCTCTCCATTC and

*OD-rev* TTCGGGTGATTCCTTGCCAC;

For luciferase assay *X. laevis* embryos were injected at 2 cells stage with 50 ng/blastomere of synthetic mRNA of Xanf1, Lanf1 or EGFP mixed with 2200-Xanf-Luc reporter plasmid^24^ and the reference pCMV-β-GAL plasmid (50 pg/embryo of each reporter plasmid). Injected embryos were collected at stage 12 in three replicate samples by 5 embryos in each and processed for luciferase analysis according to Promega protocol.

**Morpholino efficiency test**

To test efficiency of *Lanf MO1 and Lanf MO2*, *Lanf* mRNA, containing target site for the *MO* (see above), was synthesized and injected into animal pole of 4-cells *X. laevis* embryos (100 pg/blastomere) either alone or in a mixture with *Lanf MO1*, *Lanf MO2* or *misLanf MO1 MO* (6-8 nl of 0,2 mM water solution). The injected embryos were collected at the gastrula stage and analyzed for the presence of Lanf by Western blotting with Sigma Anti-His antibody as described previously^43^ (**Fig. S8b**). As one may see (**Fig. 1a** of the main text), all Lanfs contain the histidine blocks near the N-terminus. This provides a unique opportunity to detect this protein by the commercial anti-His antibodies. As a result, strong suppression of *Lanf* mRNA translation was revealed in embryos co-injected with this mRNA and any of *Lanf*-specific MO. By contrast, no inhibition of *Lanf* mRNA translation was seen in embryos co-injected with this mRNA and *misLanf MO1* (**Fig. S8b**). These results confirm the efficiency and specificity of *Lanf* MOs.

**In situ hybridization**

The chorions were removed before the fixation using sharp forceps. Embryos were fixed in MEMFA overnight at 4^0^C, dehydrated in MeOH series and stored in 100% MeOH at -20C. Before the hybridization, embryos were rehydrated, bleached (1%H2O2 in 80% MeOH overnight RT), permeabilized by proteinase K treatment (20 mkg/ml, 20 min RT), rinsed twice in 0,1 triethanolamine, pH 7,8 and acetic anhydride, washed in PBST (PBS + Triton X100 0,1%), post fixed in MEMFA (20 min RT) and washed in PBST. Pre-hybridization and hybridization buffer contain: 50% formamid, 5XSSC, 100 mkg/ml heparin, 100 mkg/ml tRNA 5mM EGTA 1% chaps , 2%Tween20. After pre-hybridization (1hour 70C), embryos were incubated overnight at 70C in hybridization buffer, containing 5mkg/ml Dig-labeled RNA probe, washed (twice with hybridization buffer, twice 2XSSC -70C, twice 0,2XSSC-RT, MAB) and incubated in blocking buffer ( MAB + 2% blocking reagent (Roch) + 20 % fetal calf serum (Sigma)) for 2 hourse RT. Then, embryos were subsequently incubated with alkaline phoshpatase (AP)-conjugated anti Dig-Fab fragment (diluted 1:1500, Roche) dissolved in blocking buffer overnight at 4C. The embryos were then washed 8 times for 60 min each in MABT buffer (MAB + 0,1% Ttiton X100) and one time overnight +4C, and soaked in AP buffer for 20 min at RT. BM purple (Roch) was used for color development.


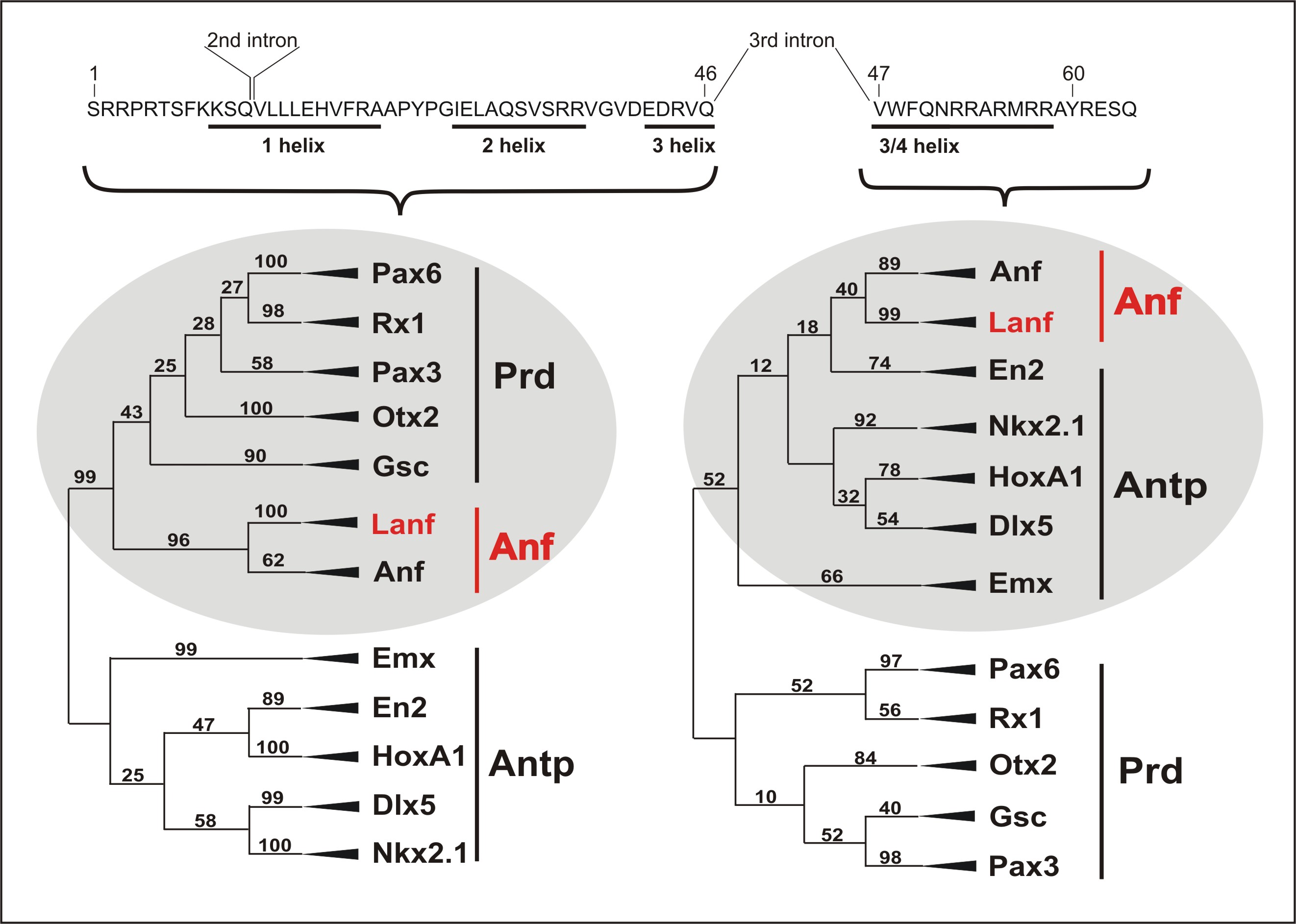


**Figure S1. Scheme of neighbor-joining^35^ phylogenetic analysis of the homology of N- and C-parts of Anf homeodomain with the corresponding regions of the Antp and Prd homeodomains (full analysis is shown on FigS5 and FigS6)**.


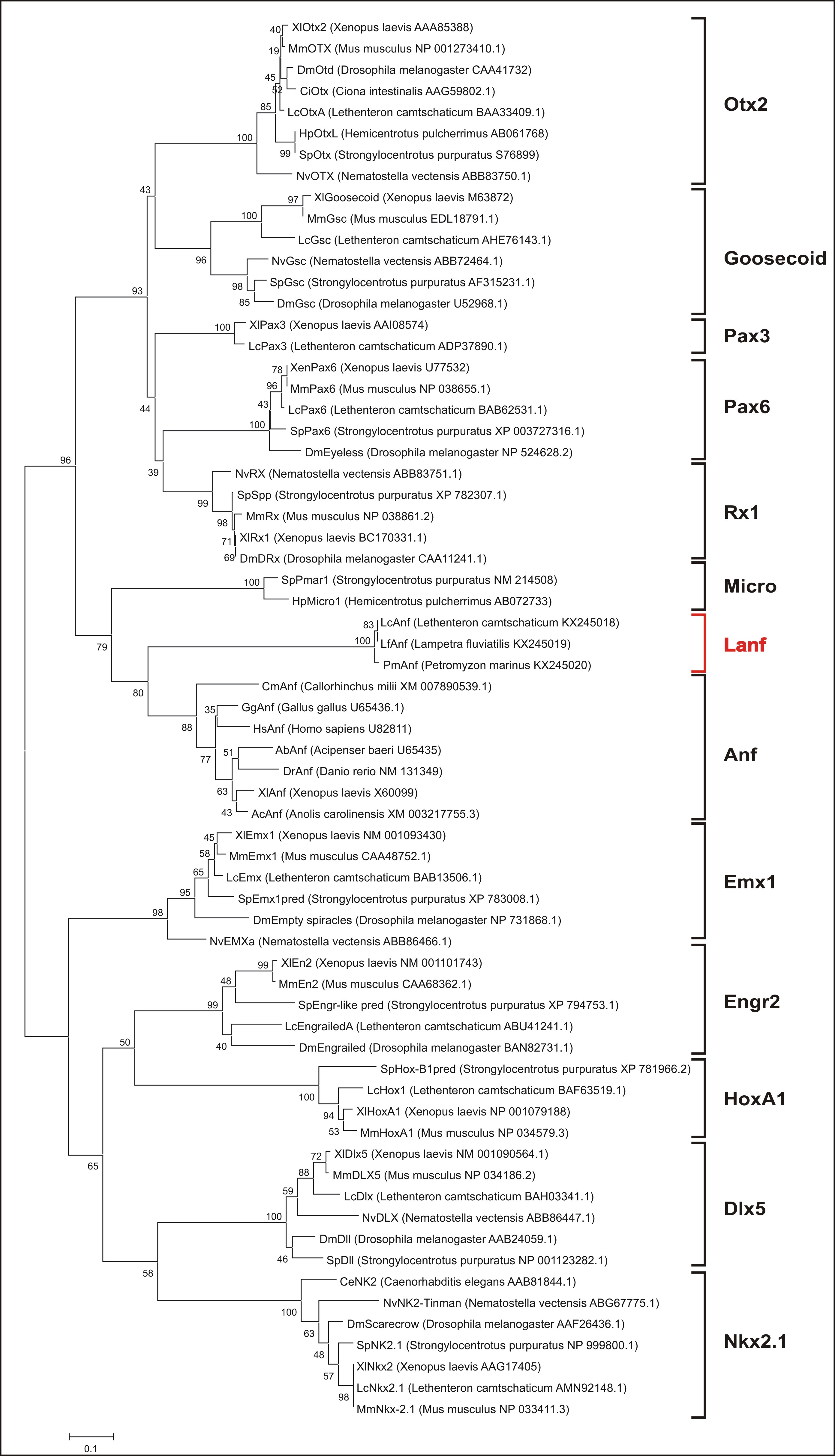


**Figure S2. Neighbor-joining^35^ tree based on 60 amino acids of the homeodomain of presented homeobox genes of different classes.**

The optimal tree with the sum of branch length = 8.92621752 is shown. The percentage of replicate trees in which the associated taxa clustered together in the bootstrap test (1000 replicates) is shown next to the branches^38^. The tree is drawn to scale, with branch lengths in the same units as those of the evolutionary distances used to infer the phylogenetic tree. The evolutionary distances were computed using the JTT matrix-based method^39^ and are in the units of the number of amino acid substitutions per site. The analysis involved 64 amino acid sequences. All positions containing gaps and missing data were eliminated. There were a total of 46 positions in the final dataset. Evolutionary analyses were conducted in MEGA6^37^. The tree was rooted by UPGMA algorithm.


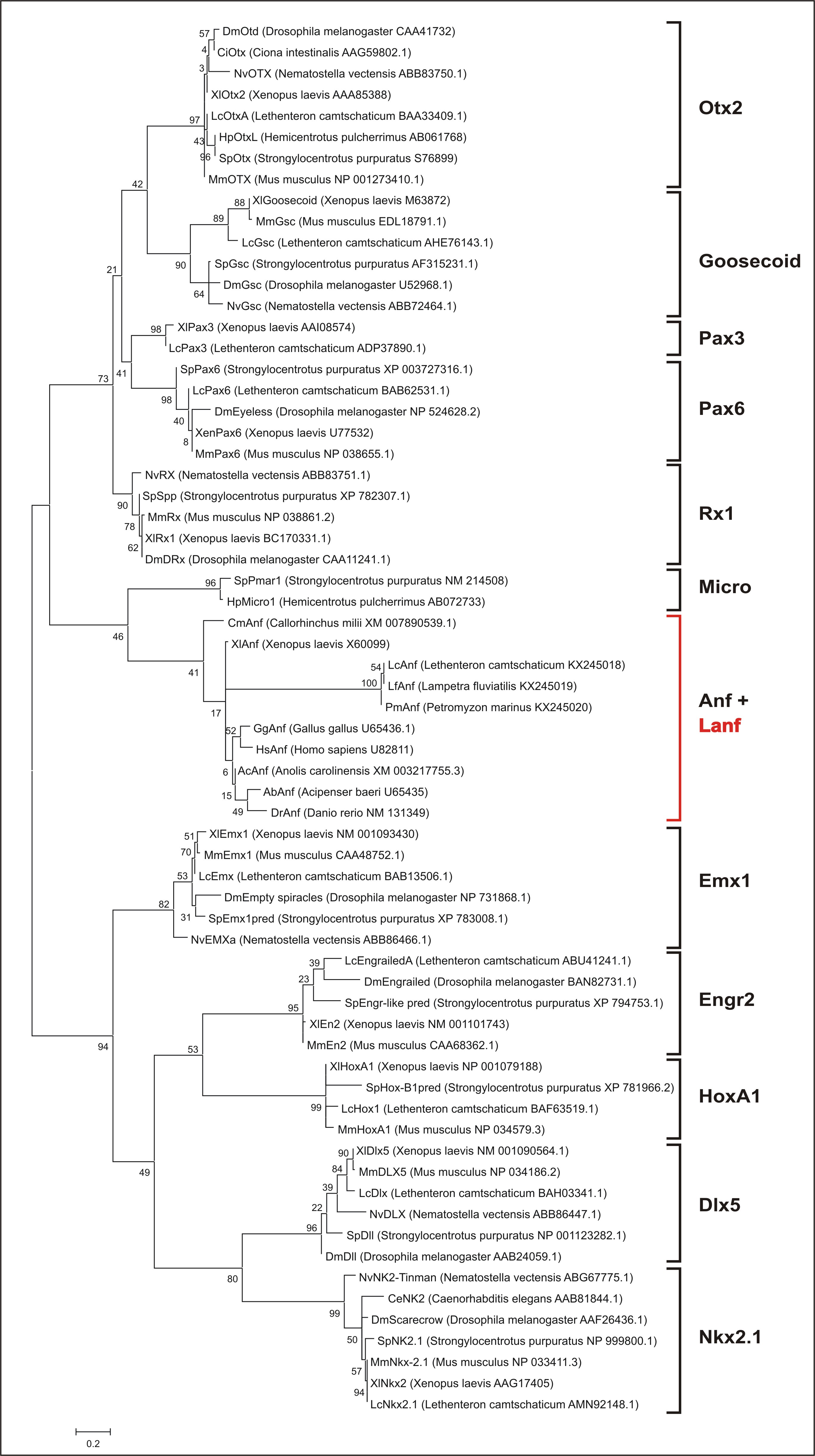


**Figure S3. Maximum likelihood tree based on 60 amino acids of the homeodomain of presented homeobox genes of different classes.**

The evolutionary history was inferred by using the Maximum Likelihood method based on the Le_Gascuel_2008 model^36^. The tree with the highest log likelihood (-2445.5349) is shown. The percentage of trees in which the associated taxa clustered together is shown next to the branches. Initial tree(s) for the heuristic search were obtained by applying the Neighbor-Joining method to a matrix of pairwise distances estimated using a JTT model. A discrete Gamma distribution was used to model evolutionary rate differences among sites (5 categories (+G, parameter = 0.6218)). The tree is drawn to scale, with branch lengths measured in the number of substitutions per site. The analysis involved 66 amino acid sequences. All positions containing gaps and missing data were eliminated. There were a total of 60 positions in the final dataset. Evolutionary analyses were conducted in MEGA6^38^. The tree was rooted by UPGMA algorithm.


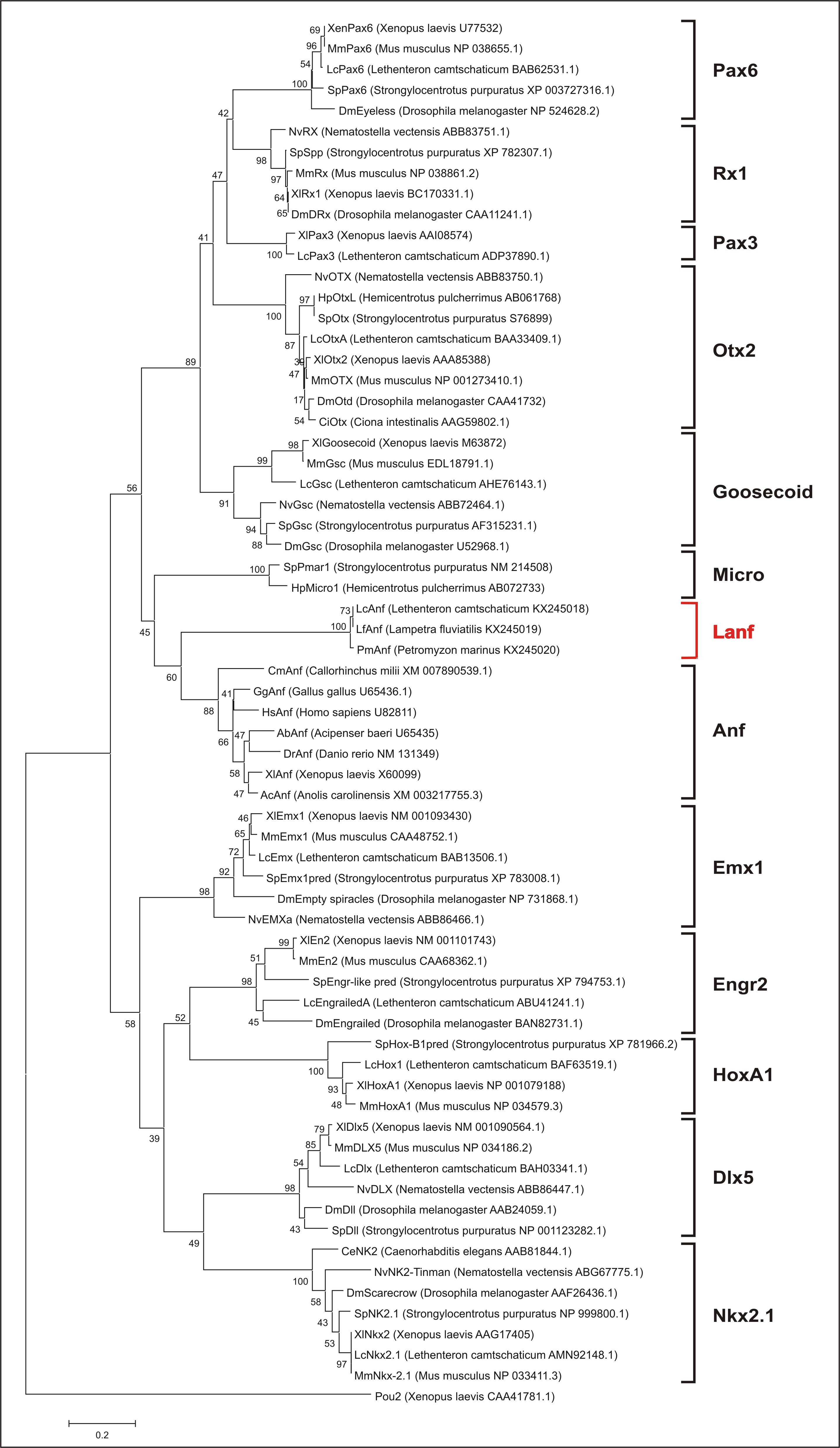


**Figure S4. Rooted neighbor-joining^35^ tree based on 60 amino acids of the homeodomain of presented homeobox genes of different classes.**

The optimal tree with the sum of branch length = 9.37197787 is shown. The percentage of replicate trees in which the associated taxa clustered together in the bootstrap test (1000 replicates) is shown next to the branches^38^. The tree is drawn to scale, with branch lengths in the same units as those of the evolutionary distances used to infer the phylogenetic tree. The evolutionary distances were computed using the JTT matrix-based method^39^ and are in the units of the number of amino acid substitutions per site. The analysis involved 67 amino acid sequences. All positions containing gaps and missing data were eliminated. There were a total of 60 positions in the final dataset. Evolutionary analyses were conducted in MEGA6^37^. The tree was rooted by including homeobox of gene PouII as the outgroup sequence.


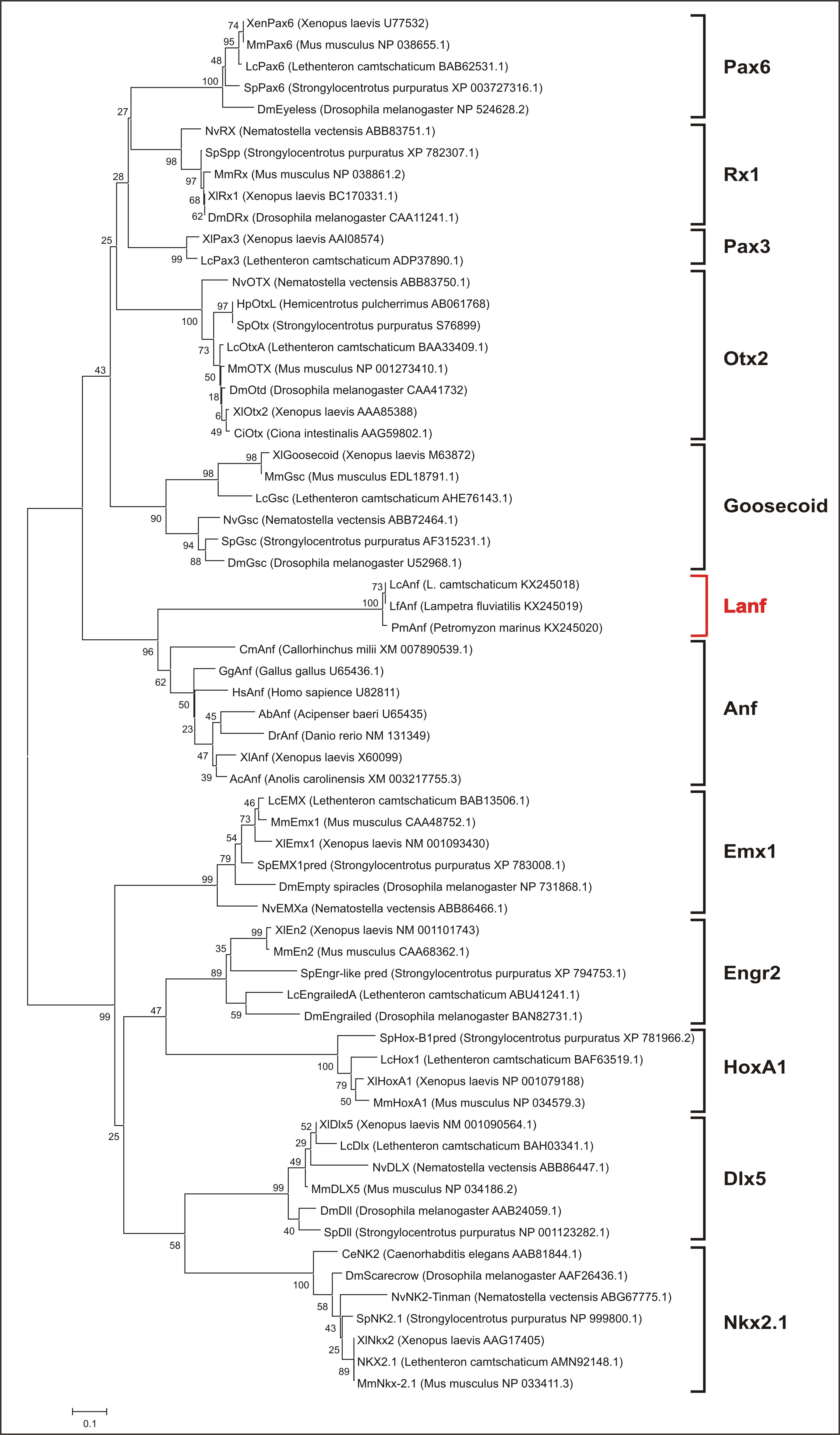


**Figure S5. Neighbor-joining^35^ tree based on 46 amino acids of the N-parts of homeodomain of presented homeobox genes of different classes (see also Figure S1).**

The optimal tree with the sum of branch length = 8.92621752 is shown. The percentage of replicate trees in which the associated taxa clustered together in the bootstrap test (1000 replicates) is shown next to the branches^38^. The tree is drawn to scale, with branch lengths in the same units as those of the evolutionary distances used to infer the phylogenetic tree. The evolutionary distances were computed using the JTT matrix-based method^39^ and are in the units of the number of amino acid substitutions per site. The analysis involved 64 amino acid sequences. All positions containing gaps and missing data were eliminated. There were a total of 46 positions in the final dataset. Evolutionary analyses were conducted in MEGA6^37^. The tree was rooted by UPGMA algorithm.


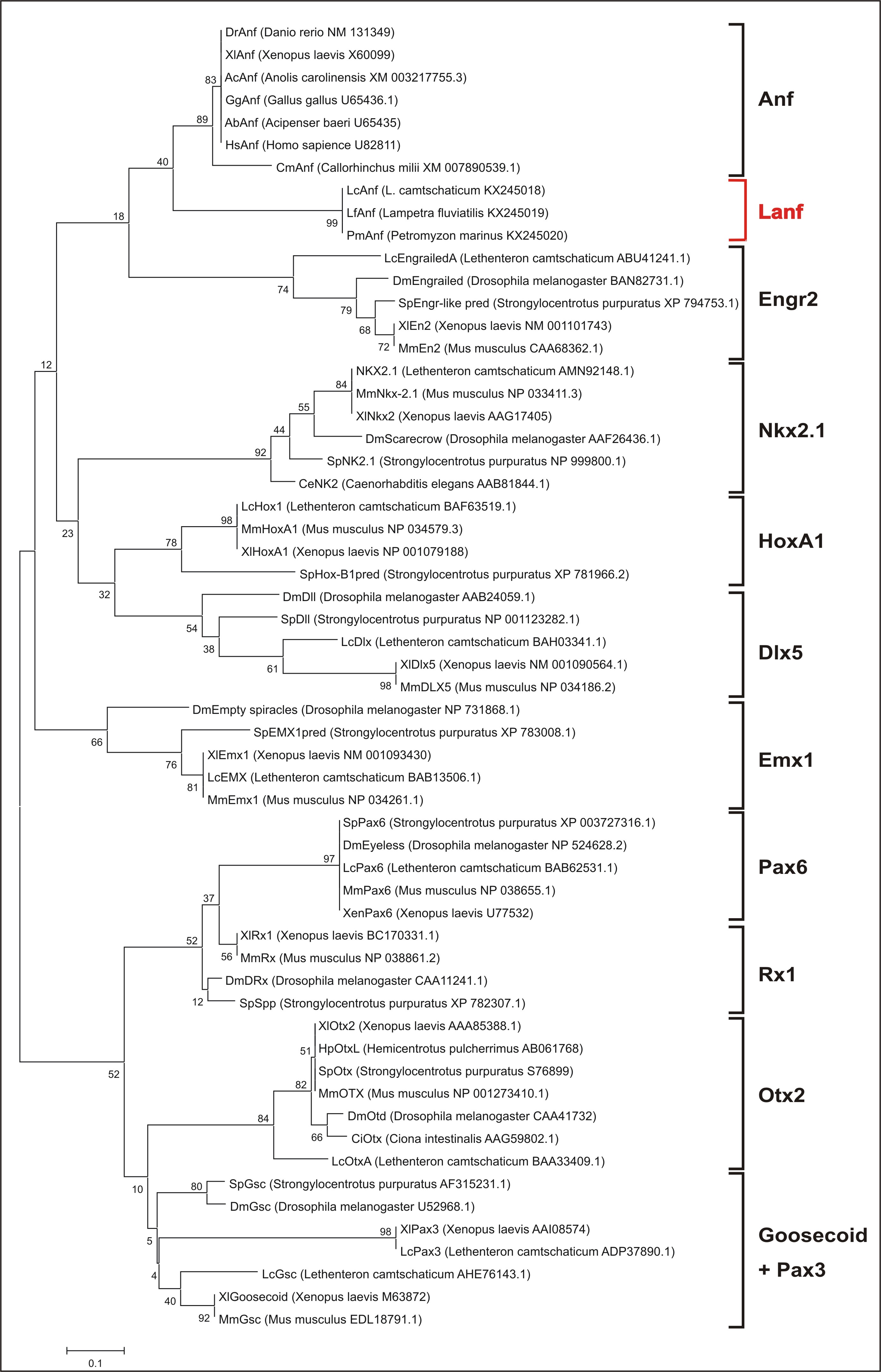


**Figure S6. Neighbor-joining^35^ tree based on 18 amino acids of the C-parts of homeodomain of presented homeobox genes of different classes (see also Figure S1).**

The optimal tree with the sum of branch length = 6.02269255 is shown. The percentage of replicate trees in which the associated taxa clustered together in the bootstrap test (1000 replicates) is shown next to the branches^38^. The tree is drawn to scale, with branch lengths in the same units as those of the evolutionary distances used to infer the phylogenetic tree. The evolutionary distances were computed using the JTT matrix-based method^39^ and are in the units of the number of amino acid substitutions per site. The analysis involved 58 amino acid sequences. All positions containing gaps and missing data were eliminated. There were a total of 17 positions in the final dataset. Evolutionary analyses were conducted in MEGA6^37^. The tree was rooted by UPGMA algorithm.


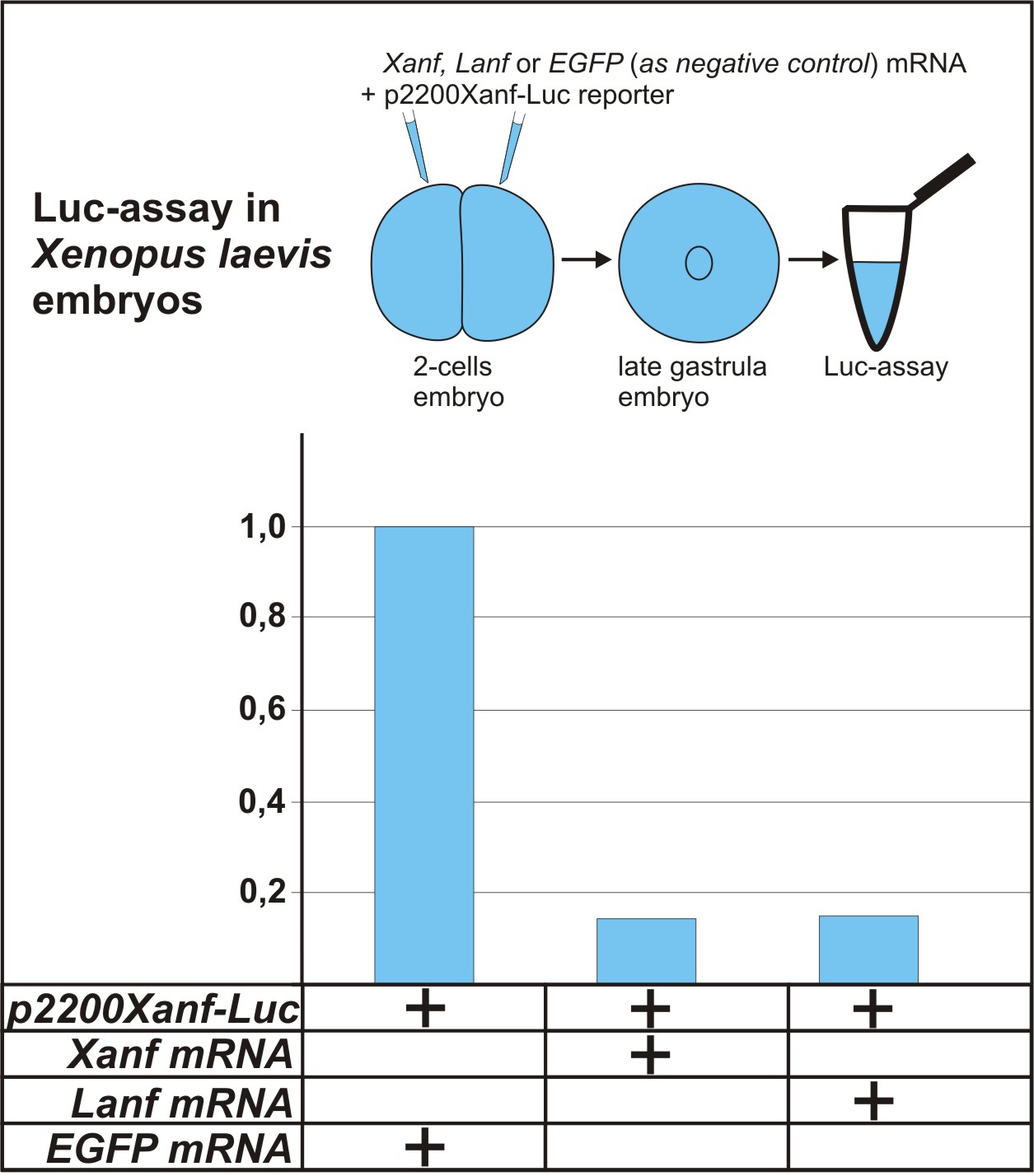


**Figure S7.** **Lanf operates as the transcriptional repressor.** Being expressed in the *X. laevis* embryos, Lanf, as well as its *Xenopus* ortholog, Xanf1, inhibits expression of the *Xanf1* promoter-driven luciferase reporter. All data from three experiments, average values are shown.

**
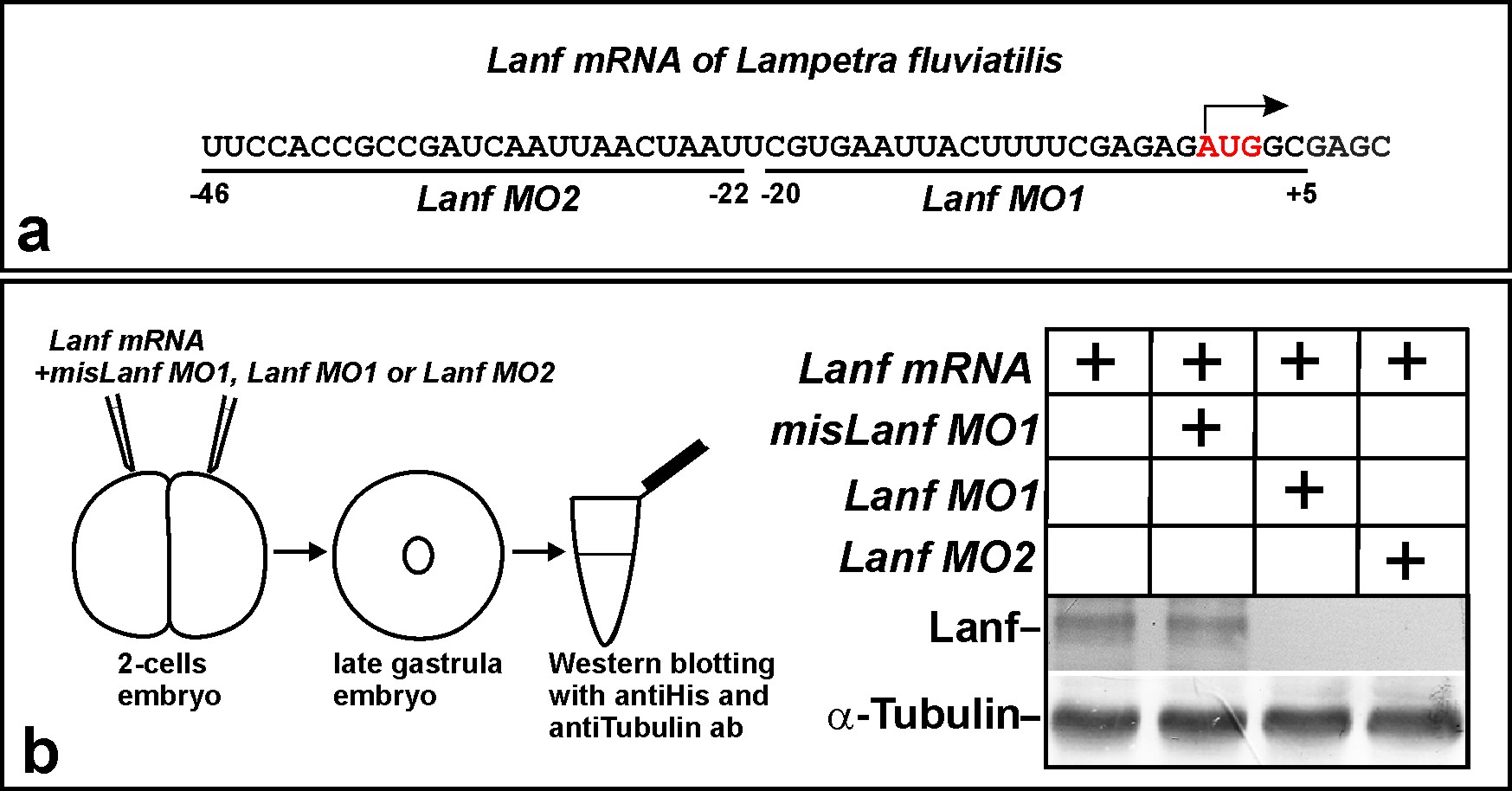
**

**Figure S8.** **Testing of *Lanf M*Os efficiency**. (**a**) *Lanf MO1* and *Lanf MO1* target sites on 5’-terminus of the *L. fluviatilis Lanf* mRNA. (**b**) Scheme and results of experiments on testing the MO efficiency. *Lanf* mRNA were injected into each blastomere of 2-cells *Xenopus laevis* embryos (100 pg/blastomere), either alone or in a mixture with control *misLanf MO1, MO1 or MO2* (8 nl of 0,2 mM water solution)*.* The injected embryos were collected at the late gastrula stage and analyzed for presence of Lanf by Western blotting with anti-His antibody (see Materials and Methods).
